# Transcription Factor Binding Sites Are Genetic Determinants of Retroviral Integration in the Human Genome

**DOI:** 10.1371/journal.pone.0004571

**Published:** 2009-02-24

**Authors:** Barbara Felice, Claudia Cattoglio, Davide Cittaro, Anna Testa, Annarita Miccio, Giuliana Ferrari, Lucilla Luzi, Alessandra Recchia, Fulvio Mavilio

**Affiliations:** 1 IFOM, FIRC Institute of Molecular Oncology Foundation, Milan, Italy; 2 IIT Unit of Molecular Neuroscience, Istituto Scientifico H. San Raffaele, Milan, Italy; 3 Cogentech, Consortium for Genomic Technologies, Milan, Italy; 4 Department of Biomedical Sciences, University of Modena and Reggio Emilia, Modena, Italy; 5 HSR-Telethon Institute of Gene Therapy, Milan, Italy; 6 Vita-Salute University, Milan, Italy; 7 Department of Experimental Oncology, European Institute of Oncology, Milan, Italy; University College Dublin, Ireland

## Abstract

Gamma-retroviruses and lentiviruses integrate non-randomly in mammalian genomes, with specific preferences for active chromatin, promoters and regulatory regions. Gene transfer vectors derived from gamma-retroviruses target at high frequency genes involved in the control of growth, development and differentiation of the target cell, and may induce insertional tumors or pre-neoplastic clonal expansions in patients treated by gene therapy. The gene expression program of the target cell is apparently instrumental in directing gamma-retroviral integration, although the molecular basis of this phenomenon is poorly understood. We report a bioinformatic analysis of the distribution of transcription factor binding sites (TFBSs) flanking >4,000 integrated proviruses in human hematopoietic and non-hematopoietic cells. We show that gamma-retroviral, but not lentiviral vectors, integrate in genomic regions enriched in cell-type specific subsets of TFBSs, independently from their relative position with respect to genes and transcription start sites. Analysis of sequences flanking the integration sites of Moloney leukemia virus (MLV)- and human immunodeficiency virus (HIV)-derived vectors carrying mutations in their long terminal repeats (LTRs), and of HIV vectors packaged with an MLV integrase, indicates that the MLV integrase and LTR enhancer are the viral determinants of the selection of TFBS-rich regions in the genome. This study identifies TFBSs as differential genomic determinants of retroviral target site selection in the human genome, and suggests that transcription factors binding the LTR enhancer may synergize with the integrase in tethering retroviral pre-integration complexes to transcriptionally active regulatory regions. Our data indicate that gamma-retroviruses and lentiviruses have evolved dramatically different strategies to interact with the host cell chromatin, and predict a higher risk in using gamma-retroviral vs. lentiviral vectors for human gene therapy applications.

## Introduction

Integration of viral cDNA into the host cell genome is an essential step in the retroviral life cycle. After entering the cell, the RNA genome is reverse transcribed into double-stranded DNA, and assembled in pre-integration complexes (PICs) containing viral as well as cellular proteins. Retroviral PICs may actively enter the nucleus of non-dividing cells, as in the case of lentiviruses (LV), or gain access to chromosomal DNA during mitosis, as in gamma-retroviruses (RV). PICs associate with the host cell chromatin, where the virally encoded integrase mediates proviral insertion into the genomic DNA [1]. Different retroviruses show significantly different integration preferences [2]–[4], implying that PICs recognize components or features of the host cell chromatin in a specific fashion [5]–[7]. Proteins interacting with the human immunodeficiency virus (HIV) integrase have been identified by biochemical or genetic analysis, and include components of the SWI/SNF chromatin-remodeling [8] or DNA-repair [9] complexes, Polycomb-group proteins [10], and lens epithelium-derived growth factor (LEDGF) [11], [12]. Much less is known about the RV integrase, and the genetic and/or epigenetic determinants of RV target site selection remain poorly understood.

Gene transfer vectors derived from the Moloney murine leukemia virus (MLV) have been used in hundreds of gene therapy clinical trials since 1991. These vectors were considered relatively safe, until lymphoproliferative disorders were reported in patients treated with MLV-transduced hematopoietic stem/progenitor cells (HSCs) for X-linked severe combined immunodeficiency (X-SCID) [13]. These adverse outcomes indicated the importance of understanding the molecular basis of retroviral integration in order to design safer gene transfer vectors [14]. The oncogenic potential of murine retroviruses has been known for decades. Administration of replication-competent retroviruses to susceptible mouse strains leads to tumor development, as a result of multiple insertion events and the outgrowth of clones containing one or more proviruses activating growth-controlling genes [15]. Replication-defective RV vectors were also reported to cause insertional oncogenesis in mice [16], but such risk was estimated to be low on the assumption that proviral integration into the genome was random [1]. Recent studies have shown that MLV-derived vectors integrate preferentially around transcription start sites (TSSs) and CpG islands [3], [4], [17]–[20], where the insertion of transcriptional enhancers contained in the viral long terminal repeats (LTRs) has a high probability to interfere with gene regulation [21]. Indeed, analysis of hematopoietic cells obtained from SCID patients treated with gene therapy showed that the vector integration characteristics increase the probability of insertional activation of proto-oncogenes [22]–[25].

Analysis of RV and LV integration sites in human HSCs showed an RV-specific propensity to integrate into hot spots and to target genes involved in the control of growth, differentiation and development of hematopoietic cells [26], [27], suggesting that the gene expression program of the target cells is instrumental in directing RV integration. This may explain the frequency by which RV integration induces activation of cell type-specific growth regulators such as LMO2 or MDS1/EVI1, and lymphoproliferative disorders in SCID patients [28], [29] or clonal expansion of hematopoietic progenitors in mice [30], [31], non-human primates [32], and man [33]. The molecular mechanisms linking RV integration to gene expression programs are, however, poorly understood. To investigate the role of transcriptional regulatory networks in directing RV and LV integration, we evaluated the local abundance and arrangement of putative transcription factor binding sites (TFBSs) in the genomic regions flanking (+/−1,000 bp) MLV and HIV proviruses. We show that RV, but not LV vectors integrate preferentially in genomic regions flanked by specific subsets of TFBSs, independently from their location with respect to genes or TSSs. Hierarchical clustering and principal components analysis of TFBS motifs flanking integration sites of different MLV and HIV mutants showed that the MLV integrase and the MLV LTR enhancer have a causal role in directing proviral integration in TFBS-rich regions of the genome. Transcription factors binding LTR enhancers in the nucleus before integration might therefore synergize with the integrase in tethering retroviral PICs to enhancer-containing domains of transcriptionally active chromatin.

## Results

### Retroviral vector integration sites in human hematopoietic cells

**Figure 1:**
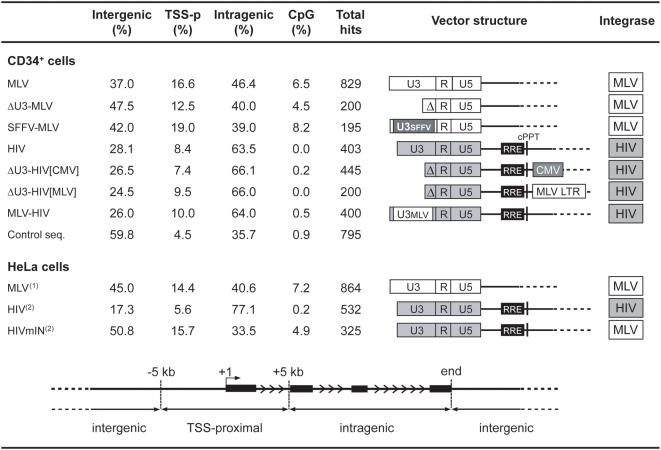
Distribution of integration sites of different RV and LV vectors identified by LM-PCR in the genome of human CD34^+^ HSCs and HeLa cells. Integration sites were annotated as ‘TSS-proximal’ when occurring within a distance of ±5 kb from the TSS of any gene, as ‘intragenic’ when occurring into a gene at a distance of >5 kb from the TSS, and as ‘intergenic’ in all other cases. The percentage of integration sites containing at least one CpG island at a distance of ±1,000 bp is also indicated (CpG %). Control sequences were randomly cloned by LM-PCR from CD34^+^ DNA samples. The structure of each vector is indicated in the middle-right panel: RV LTRs are indicated by white boxes, LV LTRs as grey boxes. U3, R and U5 regions are indicated in all LTRs. Δ indicates deletion of the U3 element. U3SFFV and U3MLV indicate the U3 elements of the spleen focus-forming virus and the Moloney leukemia virus LTR respectively. RRE, Rev-responsive element; cPPT, central polypurine tract; CMV, internal cytomegalovirus immediate-early promoter; MLV LTR, internal Moloney leukemia virus LTR. The origin of the integrase packaged with each vector is indicated in the rightmost column (MLV, white-boxed; HIV, grey-boxed). ^(1)^Original sequences from Wu *et al.* [3]. ^(2)^Original sequences from Lewinski *et al.* [19].

Human cord blood-derived CD34^+^ HSCs were transduced under cytokine stimulation with MLV-derived RV vectors carrying a wild-type LTR, a ΔU3 (enhancer-less) LTR, or an LTR from the spleen focus-forming RV (SFFV), and HIV-derived LV vectors carrying a wild-type LTR, a ΔU3 LTR or an LTR containing the MLV U3 enhancer (Figure 1). For each vector, between 195 to 829 vector-genome junctions were cloned and sequenced by linker-mediated polymerase chain reaction (LM-PCR) and mapped onto the human genome. A collection of 795 sequences randomly cloned by LM-PCR and 100,000 computer-generated random insertion sites were used as control groups. Integration sites were annotated as TSS-proximal when occurring within a distance of ±5 kb from the TSS of any Known Gene (UCSC definition), as intragenic when occurring into a gene at a distance of >5 kb from the TSS, and as intergenic in all other cases. As expected, all RV vectors showed a preference for integration around TSSs, while LV vectors integrated preferentially within genes, as compared to the control sequence set (Figure 1). Over-representation of TSS-proximal integrations was reduced in the ΔU3-MLV vector dataset (12.5% vs. 16.6 for MLV), with a concomitant, significant increase in intergenic integrations (47.5% vs. 37.0 for MLV, two-sample test for equality of proportions with continuity correction, *p*<0.01). On the contrary, similar LTR modifications (ΔU3-HIV[CMV], ΔU3-HIV[MLV] and MLV-HIV) had no apparent consequence on the LV integration preferences (Figure 1).

### Transcription factor binding sites are over-represented in sequences flanking RV integration sites

**Figure 2:**
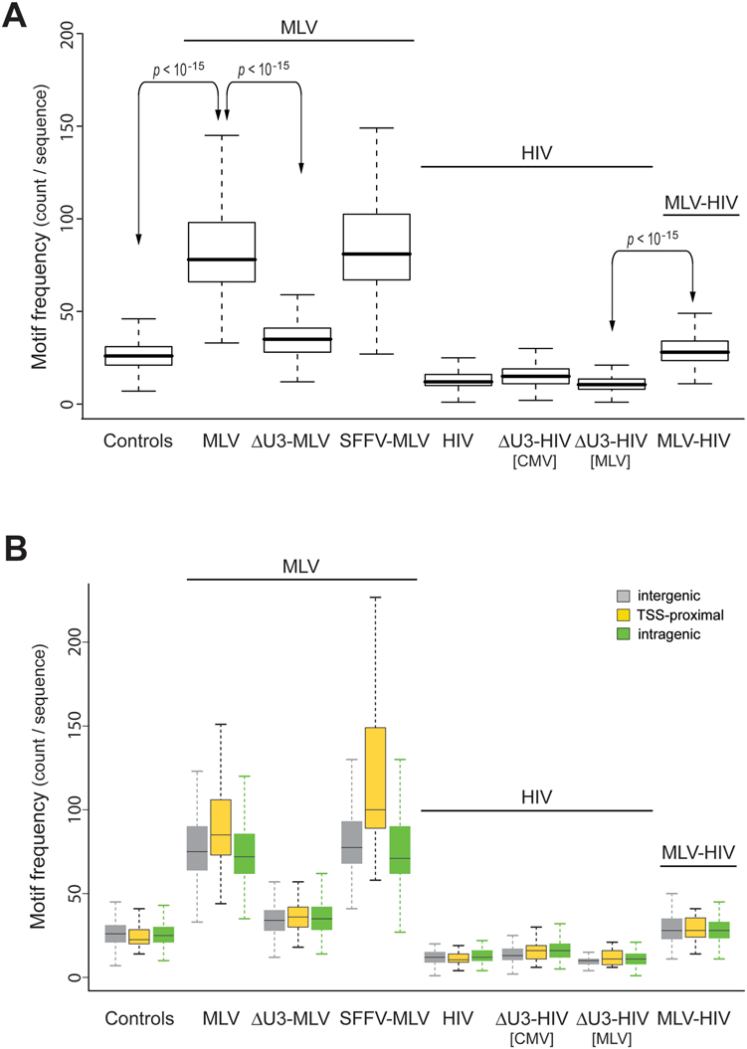
Frequency of TFBSs in genomic sequences flanking (±1.0 kb) integration sites of different RV and LV vectors (identified in Figure 1) in human HSCs. (A) Box plot of the frequency of TFBSs (motif count per sequence) in different sequence sets. Motifs derive from the JASPAR Core 2005 collection of matrix-based, non-redundant, experimentally validated TFBS motifs. Two-sample test (Wilcoxon rank sum test) statistics of the frequency comparisons among all sequence groups are reported in Table S2. *p* values of some significant comparisons are highlighted. (B) Box plot of the frequency of TFBSs (motif count per sequence) around intergenic (grey), TSS-proximal (yellow), and intragenic (green) integrations.

To investigate the role of transcription in mediating retroviral target site selection, we evaluated the abundance of transcription factor binding sites (TFBSs) in a ±1,000-bp interval from the integration sites of the RV and LV vectors in human HSCs. Based on the annotation reported in Figure 1, we generated seven weighted control groups of random sequences that reproduce, in proportion, the integration preferences of each vector set (Table S1). These sequences were used as pair-weighted background to analyze the frequency of TFBS around insertion sites by the Clover program, which screens DNA sequence sets against a precompiled library of motifs and provides statistically significant over- or under-representation compared to a background set of sequences [34]. For this analysis, we used the JASPAR Core 2005 database, an open-access database of 123 annotated, matrix-based TFBS motifs for multicellular eukaryotes [35]. Compared to other databases (e.g., TRANSFAC), JASPAR motifs are non-redundant and are derived exclusively from sets of nucleotide sequences experimentally demonstrated to bind TFs. The number of motifs enriched in each group of sequences with respect to its fitted background is plotted in Figure 2. In all groups, motifs were uniformly distributed through the ±1,000-bp window (data not shown), which was chosen as a reasonable compromise between amount of information and heaviness of computation. The box plots in Figure 2A indicate that RV vectors integrate in genomic regions highly enriched in TFBSs (86.8 and 90.3 average TFBS count per sequence for MLV and SFFV-MLV respectively vs. 27.2 for control sequences, Wilcoxon rank sum test, *p*<2.2e-16; complete statistics in Table S2). The enrichment is independent from the relative position of integration sites with respect to genes and TSSs, since it is present in intergenic as well as in TSS-proximal and intragenic integrations (grey, yellow and green box plots respectively in Figure 2B). The RV LTR enhancer appears to play an essential role in this selection, since deletion of the U3 region, but not its replacement with the SFFV enhancer, causes a significant drop in the abundance of TFBSs around the integration sites (35.4 for ΔU3-MLV vs. 86.8 for MLV). Conversely, sequences around LV vector integration sites show a significantly lower TFBS content compared to control sequences, (12.8 vs. 27.2). Interestingly, replacement of the HIV U3 by the MLV U3 enhancer in the HIV LTR (MLV-HIV vector in Figure 2) appears to bias LV integration towards regions with an increased content of TFBSs (from 12.8 of HIV to 29.1 of MLV-HIV, *p*<2.2e-16; complete statistics in Table S2). The MLV U3 enhancer plays this role only in the context of the LTR, as it has no apparent effect when placed in an internal position within the LV vector (ΔU3-HIV[MLV] vector in Figure 2).

Sequences flanking retroviral integration sites were tested also for the presence of CpG islands. As expected from previous reports [19], CpG island were enriched in all RV sequences with respect to controls, while they were under-represented or completely absent in LV sequences (Figure 1). Over-representation of CpG islands was reduced in the ΔU3-MLV vs. the MLV and SFFV-MLV dataset (4.5% vs. 6.5 and 8.2%), suggesting a role for the U3 enhancer in targeting CpG island-containing regions. Despite the enrichment in CpG islands, the GC content of the RV vector sequences was comparable to that of the random control sequences (44.4% for MLV, 44.6% for SFFV-MLV and 44.7% for ΔMLV, vs. 43.7% for random sequences). On the contrary, the GC content of LV sequences was significantly lower than controls and unaffected by LTR modification (37.6% for HIV, 38.1% for ΔU3-HIV[CMV], 38.8% for ΔU3-HIV[MLV] and 38.2% for MLV-HIV vs. 43.7% for random sequences).

### Retroviral integration sites are flanked by vector-specific patterns of transcription factor binding sites

**Figure 3:**
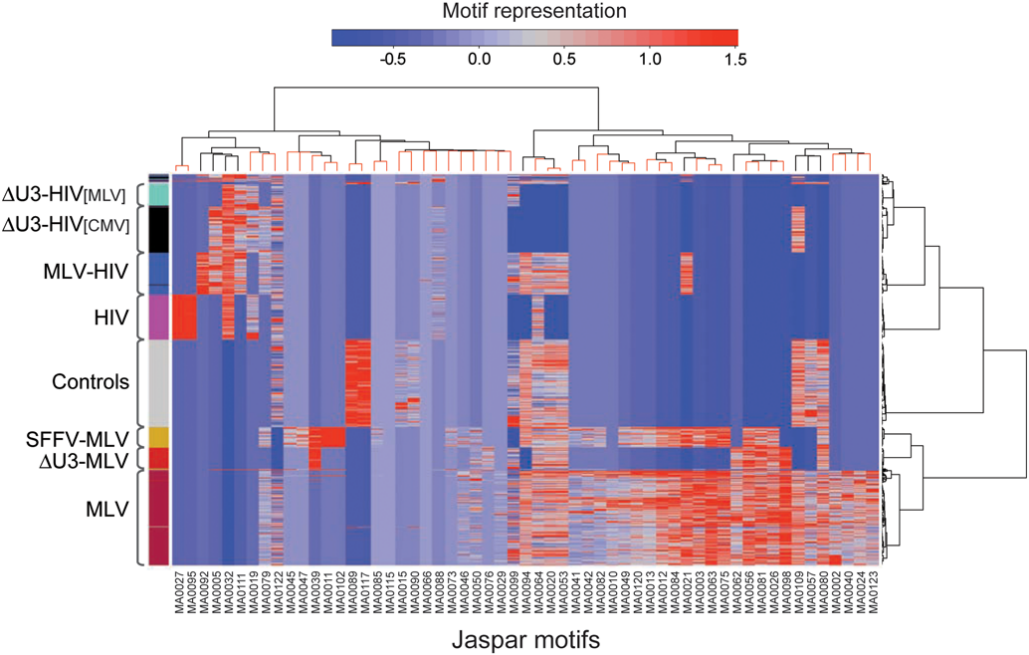
Unsupervised, two-way hierarchical cluster analysis of the relative frequency of TFBS motifs around integration sites of different RV and LV vectors (identified in Figure 1) in human HSCs. The heatmap, computed with likelihood ratio values derived from the Clover analysis of motif representation, indicates the relative frequency by which each motif (columns) is represented in each sequence (rows) (red, over-representation; blu, under-representation). Motifs are identified by the JASPAR ID at the bottom (complete list in Table S3). The row dendrogram (right) identifies three main branches corresponding to MLV, Control and HIV sequences. The bootstrapped column dendrogram (top) splits the dataset in two main branches, segregating RV from LV and Control sequences. Red branches on the tree identify “stable” nodes with an Approximately Unbiased (AU) test *p*-value>0.95 (detailed dendrogram in Figure S1).

To identify TFBS motifs specifically associated with the different sets of sequences, we performed an unsupervised, two-way hierarchical clustering of the relative frequency of each motif (likelihood ratio values) obtained from the Clover analysis. The associations are graphically represented in the heatmap in Figure 3, where the color grading indicates the frequency by which each motif (columns) is represented in each sequence (rows). The unsupervised analysis clusters together with remarkable precision sequences belonging to the same datasets, indicating that the integration sites of different vectors are defined by specific patterns of flanking TFBS motifs. The row dendrogram (right) identifies three main nodes corresponding to RV, control and LV sequences, which originate secondary branches identifying the different vector designs. To add robustness to the analysis, we applied an approximately unbiased (AU) test on column dendrograms, sampling them with 10,000 multiscale bootstrap replicates [36]. Nodes having an AU *p*-value>0.95 were scored as significant (stable) nodes, and are represented by red branches in Figure 3 (complete analysis in Figure S1). The bootstrapped column dendrogram (top) splits the dataset in two major branches, defining LV and RV vector profiles. A core of four motifs (MA0056, MA0081, MA0026, MA0098) is strongly associated (AU = 100) to all MLV vectors, independently from their LTR structure. Three of these motifs (MA0081, MA0026, MA0098) are bound to TFs belonging to the ETS family, and one (MA0056) to TFs of the Zn-finger C_2_H_2_ family. Interestingly, sequences flanking the integration sites of the enhancer-less LTR vector (ΔU3-MLV) lack a set of 12 motifs common to MLV and SFFV sequences, and 5 motifs common to MLV sequences only. These motifs are therefore associated to an RV or specifically to the MLV U3 enhancer. All JASPAR motifs are identified in Table S3, which lists their frequency in each sequence set.

The hierarchical cluster analysis confirms a strong under-representation of TFBSs in all HIV sequences, which shared only one characterizing forkhead motif (MA0032). Although the insertion of the MLV U3 region in the HIV LTR increased the absolute TFBS motif count around integration sites (Figure 2), it was not sufficient to change the segregation of the MLV-HIV vector sequences in the cluster analysis. Figure 3 shows that the MLV-HIV sequences share most of their motif profile with LV sequences, with the notable exception of one Zn-finger motif (MA0021) that is shared instead with the MLV and SFFV-MLV vectors.

**Figure 4:**
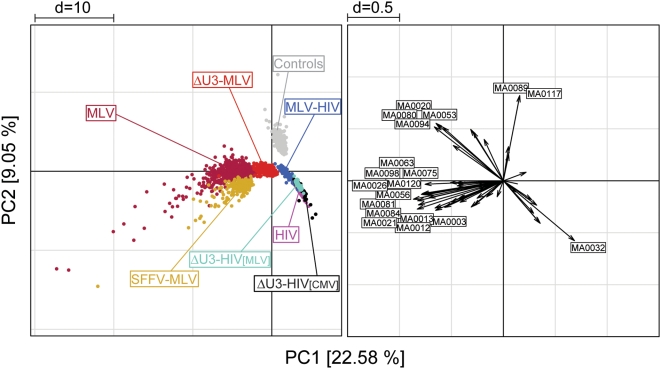
Principal component analysis of likelihood ratio values from the Clover analysis for 57 enriched TFBS motifs. A scatter plot of the first two components, accounting for 31.6% of the total variability (left panel), shows three main groups: RV sequences (MLV, SFFV-MLV and ΔU3-MLV), LV sequences (HIV, ΔU3-HIV[CMV], ΔU3-HIV[MLV], and the hybrid MLV-HIV), and Control sequences. The first component (x-axis) discriminates between RV and all other sequences, the second component (y-axis) between LV and Control sequences. ΔU3-MLV sequences, containing a lower number of TFBSs, show less variability than the MLV and MLV-SFFV sequences, but are still oriented towards the RV group along the first component axis. A plot of 19 loading vectors having a value higher than the chosen cutoff (right panel) shows one vector (motif ID: MA0032) oriented with the LV group, two (MA0117 and MA0089) with the Control group, and the remaining ones with the RV group. The four motifs (MA0056, MA0081, MA0026 and MA0098) strongly associated with RV sequences in the cluster analysis (AU values = 100) are contained in this group. All motifs are identified in Figure 5.

**Figure 5:**
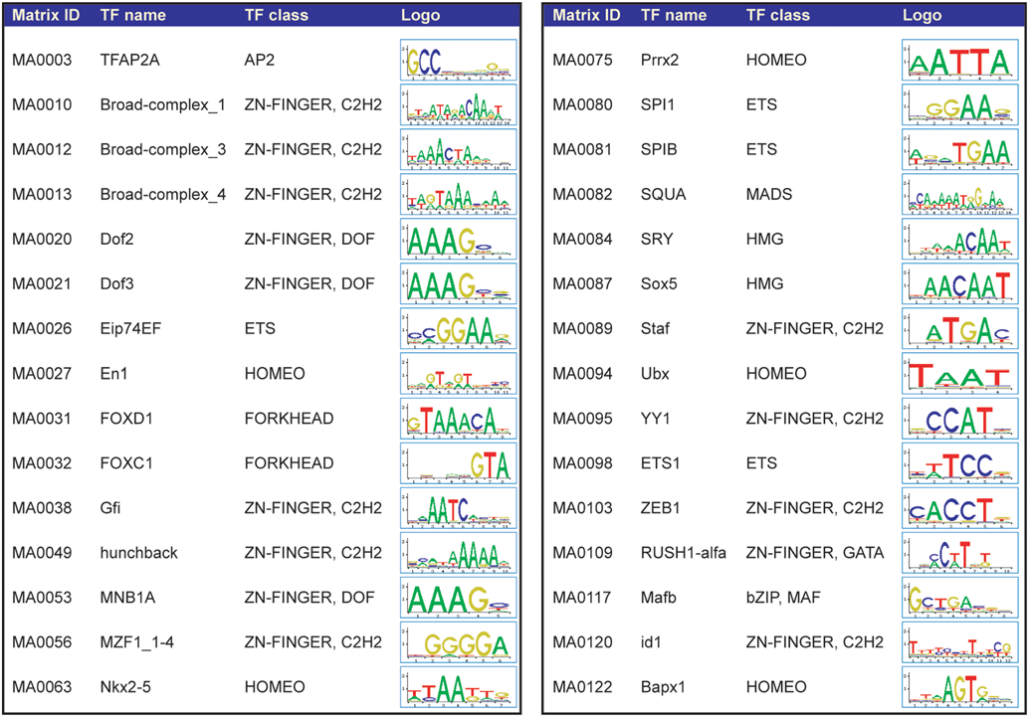
Summary table of all over-represented TFBS motifs emerging from PCA analyses reported in Figures 4, 7 and 9. For each motif, identified by its JASPAR ID, the table specifies the name of the associated transcription factor (TF), the class to which the TF belongs, and the relative consensus sequence (Logo).

The results of the cluster analysis were independently confirmed by a principal component analysis (PCA), a technique that identifies simultaneously all the existing correlations between samples and variables in multivariate data sets, and orders them according to their contribution to the total variance of the system. The PCA transforms a number of possibly correlated variables, i.e., TFBS motifs, into a smaller number of uncorrelated variables called principal components (PCs). A scatter plot of the first two components, accounting for 31.6% of the total variability, identifies three main groups: RV sequences (MLV, SFFV-MLV and ΔU3-MLV), LV sequences (HIV, ΔU3-HIV[CMV], ΔU3-HIV[MLV], and the hybrid MLV-HIV), and control sequences (Figure 4). The first component discriminates between RV and all other sequences, the second one between LV and control sequences, oriented in opposite direction along the second component axis (left panel). The variability within MLV and SFFV-MLV data is higher that in any other group, possibly because of the high number of TFBSs contained in those sequences. ΔU3-MLV sequences contain a lower number of TFBSs and show a lower variability, although they are still oriented towards the RV group along the first component axis. The loading plot on the right panel shows a high number of motifs (represented as vectors) contributing to the RV group. Among the 19 loadings with a length higher than the chosen cutoff, one (MA0032) is oriented with the LV group, two (MA0117, MA0089) with the control group, and the remaining ones with the first principal component. Twelve of these vectors are exclusively oriented with the RV group, and belong to different TFBS families: four motifs are recognized by Zn-finger C_2_H_2_, three by ETS, two by homeodomain-containing, and one by Zn-finger-dof, HMG, and AP2 transcription factors. Interestingly, this group contains the four motifs strongly associated with RV sequences in the cluster analysis (MA0056, MA0081, MA0026, and MA0098 in Figure 3). All motifs identified by the loading plot are listed in Figure 5, which shows their consensus sequences and their associated transcription factors.

### Evolutionarily conserved TFBSs are enriched in sequences flanking RV integration sites

**Figure 6:**
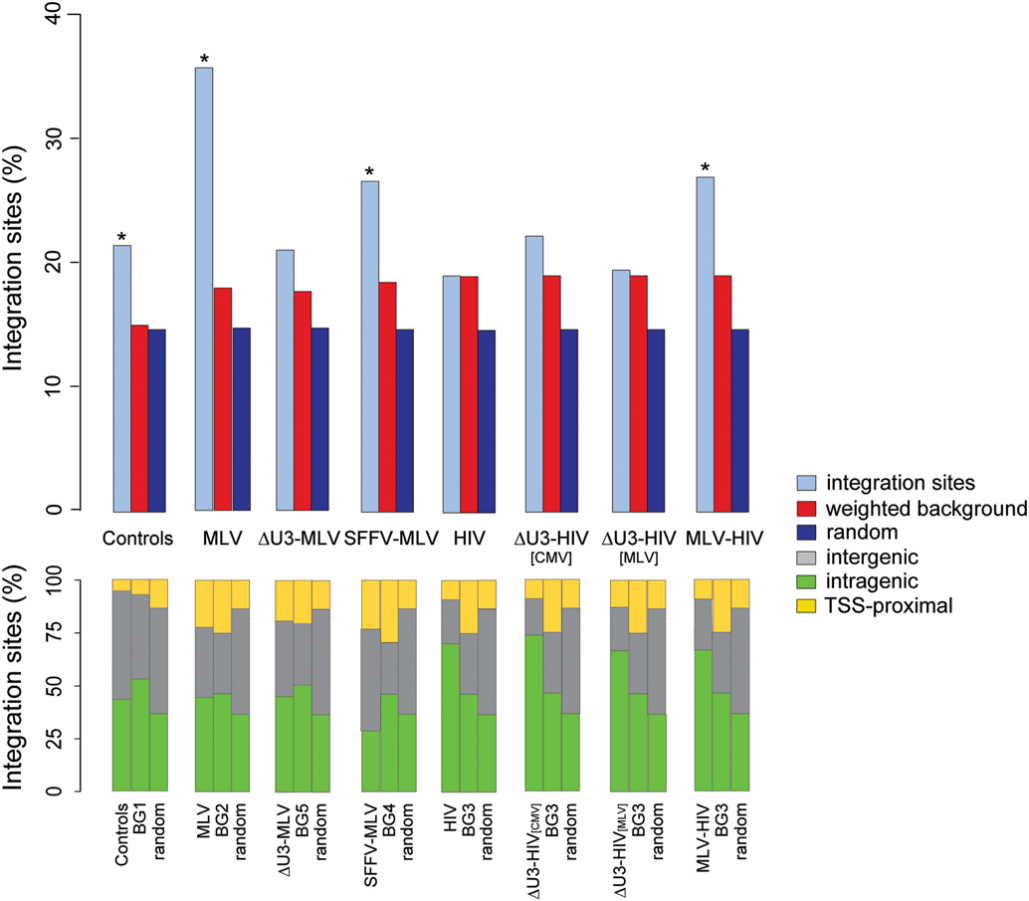
Analysis of the frequency of evolutionarily conserved TFBSs in the sequences flanking the integration sites of different RV and LV vectors (identified in Figure 1) in human HSCs. Motifs derive from the TFBS Conserved Track at the UCSC Genome Browser, which includes 188 motifs from the TRANSFAC Matrix Database (v 7.0) conserved in a human-mouse and/or -rat genome alignment. In the upper panel, data are plotted as percentage of sequences containing at least one conserved motif. Each group of sequences (light blue bars) is compared to a weighted background (BG, red bars) and a random computational control sequence set (blue bars) (see methods for definitions). Asterisks highlight experimental groups that show a significant enrichment of frequency compared to control sets (one-sided Fisher test; complete statistics in Table S4). In the lower panel, frequency data are broken down into three subgroups according to the integration site annotation, i.e., intergenic (gray bars), TSS-proximal (yellow bars), and intragenic (green bars). The complete list of conserved motifs and their distribution in the different datasets are reported in Table S5.

A significant over-representation of TFBSs was observed around RV integrations also when considering only evolutionarily conserved binding sites. For this analysis, we used the TFBS Conserved Track at the UCSC Genome Browser, which includes 188 motifs from the TRANSFAC Matrix Database (v 7.0) conserved in a human-mouse and/or -rat genome alignment. A total motif count was determined for each experimental and control sequence, and a Fisher exact test (two-sided, confidence level = 0.95) was used to determine statistical significance. The complete list of conserved motifs and their distribution over the different datasets are reported in Table S5. The bar plot in Figure 6 (upper panel) shows that 35.7% and 26.7% of the sequences flanking MLV and SFFV-MLV integration sites respectively contained at least one conserved TFBS (range: 2–30 sites/sequence), a significant difference with respect to their weighted backgrounds and to a random computational control set of 100,000 sequences (17.9, 18.5 and 14.7% respectively) (Figure 6, upper panel). Sequences flanking the ΔU3-MLV and all HIV integration sites showed no significant enrichment, again with the exception of the MLV-HIV hybrid vector (complete statistics in Table S4). Intragenic, intergenic and TSS-proximal sequences contributed proportionally to the conserved TFBS over-representation in all samples (Figure 6, lower panel). Given the tight constrains in the definition, conserved TFBSs were scored in much smaller numbers than in the Clover analysis. The motifs consistently associated with MLV integration by both analyses are listed in Table 1. These motifs are predicted to bind homeodomain, ETS, bZIP, forkhead and Zn-finger proteins, including the cell-type specific growth regulators AML1/RUNX1, FOXO3 and LMO2.

**Table 1:** TFBS motifs found significantly enriched in sequences flanking (±1,000 bp) the integration sites of the MLV vector in human HSCs in both the JASPAR and the TRANSFAC conserved motif database.

JASPAR					TRANSFAC (conserved)		
Matrix ID	TF	Total counts	Counts/seq (average)	Counts/seq (range)	Matrix AccNumb	TF	Total counts
MA0109	Rush 1α	530	0.63	0–3	M00278	LMO2	18
MA0046	TCF1	871	1.05	0–5	M00132	HNF1	12
MA0002	RUNX1	1,146	1.38	0–4	M00454	MRF2	16
MA0050	IRF-1	1,463	1.76	0–6	M00062	IRF-1	20
MA0012	broad complex_3	1,531	1.84	0–12	M00474	FOXO1	30
MA0123	ABI4	1,726	2.08	0–10	M00515	PPRG	6
MA0026	E74A	1,940	2.34	0–7	M00025	ELK1	4
MA0064	PBF	2,028	2.44	0–9	M00062	IRF-1	20
MA0042	FOXI1	2,217	2.67	0–11	M00289	FOXI1	8
MA0053	MNB1-A	2,246	2.70	0–9	M00062	IRF-1	20
MA0013	broad complex_4	2,297	2.77	0–20	M00477	FOXO3	30
MA0120	Id1	2,553	3.07	0–21	M00258	ISGF3	20
MA0079	Sp1	2,648	3.19	0–10	M00257	RREB1	6
MA0021	dof3	2,902	3.50	0–10	M00062	IRF-1	20
MA0020	dof2	3,201	3.86	0–10	M00062	IRF-1	20

Frequencies are listed as total counts, average counts per sequence, and range of counts per sequence (1^st^ to 99^th^ percentile) in the 829 MLV sequences. JASPAR and TRANSFAC motifs were matched by the STAMP software [55].

### Patterns of TFBS motifs flanking retroviral integration sites are cell-type specific

**Figure 7:**
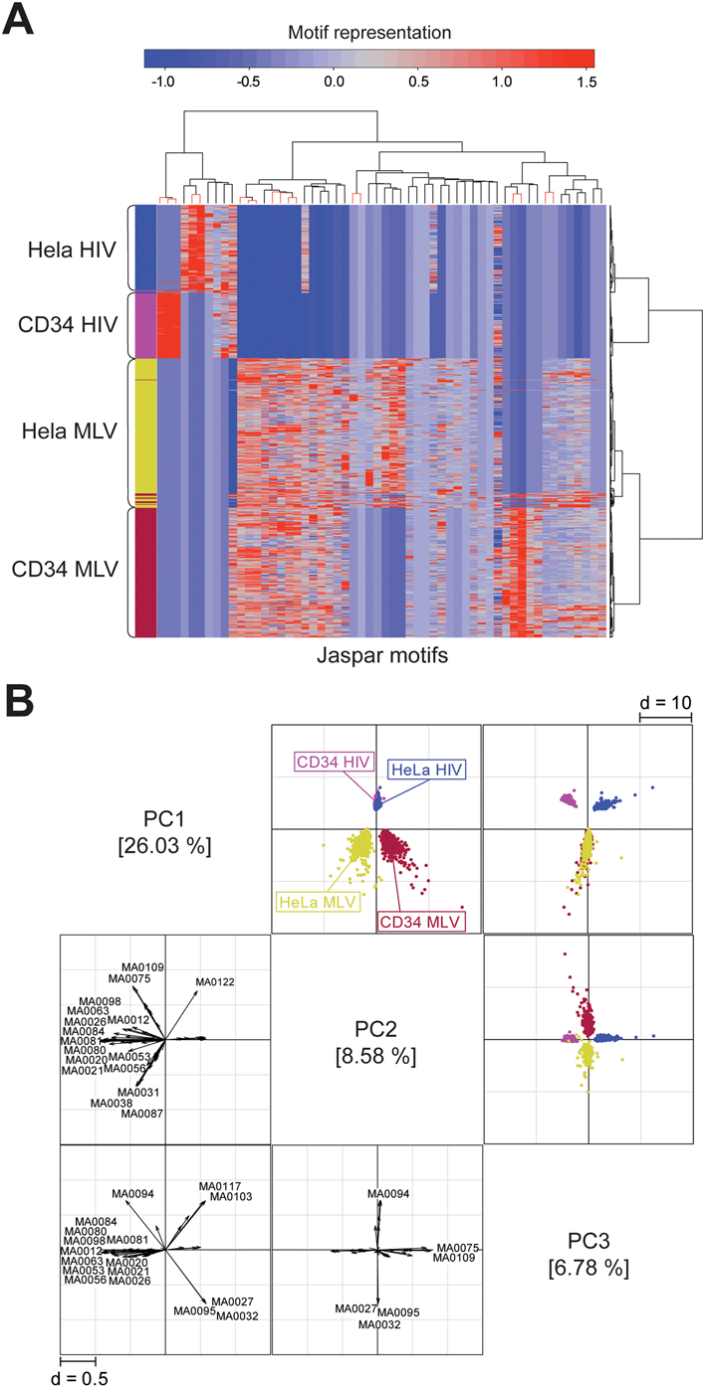
Frequency and distribution of TFBSs in genomic sequences flanking integration sites (+/−1.0 kb) of RV and LV vectors (identified in Figure 1) in HSCs and HeLa cells. (A) Two-way hierarchical cluster analysis (see Figure 3 for definitions). The row dendrogram (right) splits the dataset in two branches (MLV and HIV), within which HSC and HeLa sequences are clearly separated. The bootstrapped column dendrogram (top) split the cluster in two nodes, mainly related to the HIV and the MLV profile (detailed dendrogram in Figure S1, complete list of motifs in Table S3). (B) Principal component analysis of likelihood ratio values from the Clover analysis. The scatter plots (upper-right, colored squares) of the first three principal components, accounting for 41.4% of the total variability, and the corresponding loading plots (lower-left, b/w squares) were combined. On the scatter plots, the first source of variability is the vector type: MLV and HIV sequences distribute on the first component in opposite direction. The second and third sources of variability are the cell context within MLV and HIV sequences respectively. The loading plots show that motifs that better explain this specific behavior are the same identified in the hierarchical cluster analysis (panel A and Figure S1) Motifs are identified in Figure 5.

To understand whether the cell context has a role in targeting retroviral integration, we compared the sequences flanking MLV and HIV integration sites in CD34^+^ cells with sequences obtained from published collections of retroviral integration sites in the human epithelial cell line HeLa [3], [19] (Figure 1). Also in these cells, MLV vectors integrate in TFBS-rich regions compared to HIV vectors (Figure S2). A two-way hierarchical cluster analysis showed cell type-specific as well as common sets of over-represented motifs (Figure 7A). The row dendrogram (right) splits the whole dataset in two branches (MLV and HIV), within which HSC and HeLa sequences are clearly separated. The bootstrapped column dendrogram (top) splits the matrix dataset in two main nodes, defining RV and LV distinct patterns (complete dendrogram with AU values for each node is reported in Figure S1). The cluster analysis shows that three Zn-finger (MA0021, MA0020, MA0053), four ETS (MA0081, MA0026, MA0080, MA098) and two forkhead (MA0041, MA0042) motifs are strongly associated (AU *p*-value>0.95) with MLV sequences in both cell types. On the contrary, two bHLH-ZIP motifs (MA0058, MA0059) are associated only with HeLa cells and two Zn-Finger GATA motifs (MA0075, MA0109) with HSCs. Among HIV sequences, three motifs are associated with HSCs (MA0095, MA0027 and MA0032), and two (MA0103 and MA0117) with HeLa cells (Figures 7A and S1).

A PCA confirmed the results obtained by the cluster analysis. A scatter plot of the first three principal components, accounting for 41.4% of the total variability, confirms the vector type as the first source of variability (Figure 7B). The second and third components segregate the cell context (HSC vs. HeLa) within MLV and HIV sequences respectively (Figure 7B). The corresponding loading plots show that motifs that better explain the variability are the same identified in the hierarchical cluster analysis (Figure 7B). All motifs identified in the loading plot are shown in Figure 5.

### The MLV integrase has a crucial role in directing retroviral integration in TFBS-rich regions of the genome

**Figure 8:**
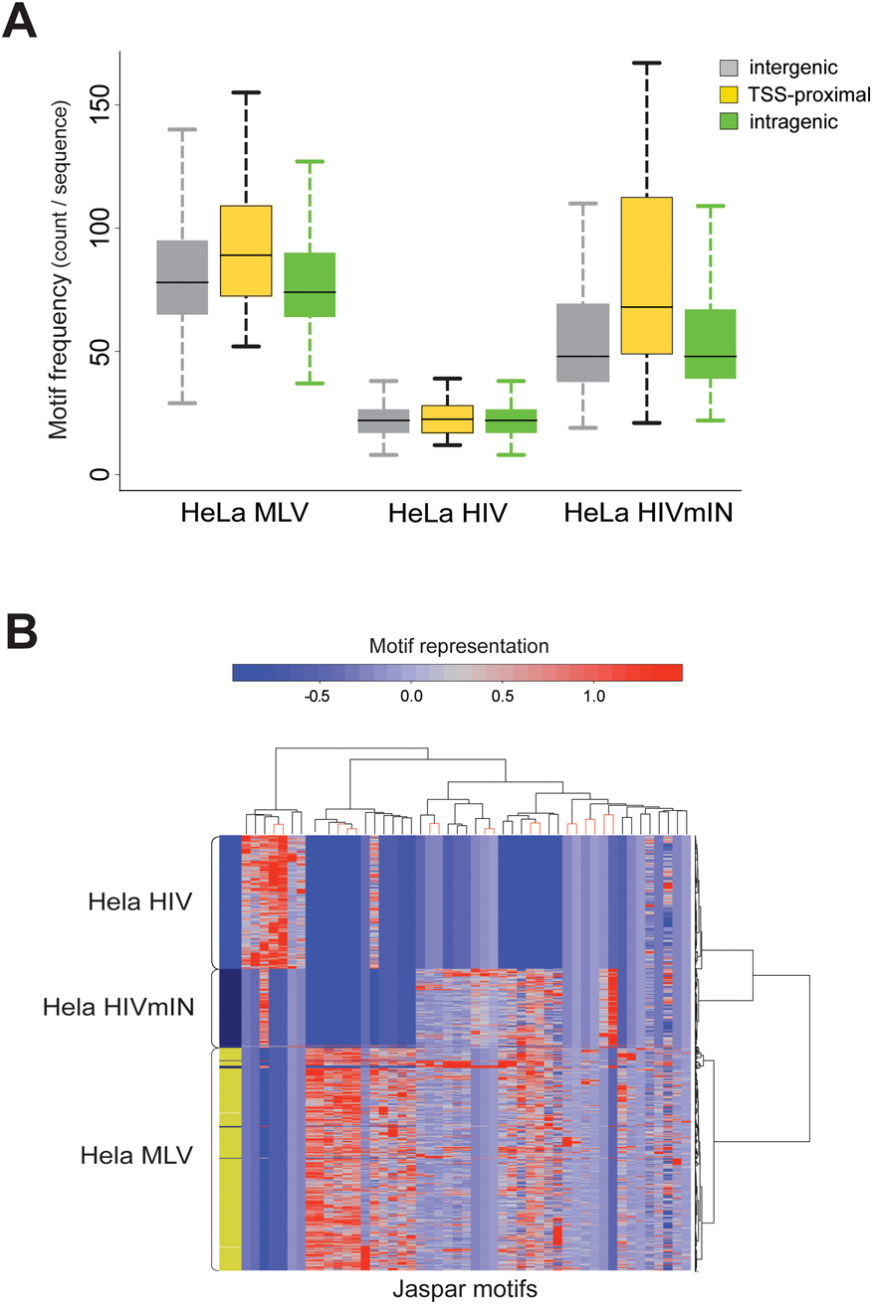
Analysis of the role of the MLV integrase in retroviral target site selection. (A) Box plot of the frequency of TFBS motif from the JASPAR database (motif count per sequence) around intergenic, TSS-proximal, and intragenic integration sites in HeLa cells of an MLV vector, an HIV vector, and an HIV vector packaged with an MLV integrase (HIVmIN) (vectors are identified in Figure 1). Two-sample test (Wilcoxon rank sum test) statistics of the frequency comparisons among and within groups is reported in Table S2. (B) Two-way hierarchical cluster analysis (see Figure 3 for definitions). The row dendrogram (right) clearly separates MLV and HIV sequences. TFBSs are under-represented in HIV sequences compared to MLV sequences, while sequences from the HIVmIN vector share a 7–motif branch with those of the MLV vector in the column dendrogram (detailed dendrogram in Figure S1, complete list of motifs in Table S3).

A recent study indicated that the MLV integrase has a crucial role in determining the RV characteristic preference for TSS-proximal regions and CpG islands [19]. To provide evidence for a role of the MLV integrase in directing integration to TFBS-rich regions, we carried out a comparative analysis of the sequences flanking the integration sites of an MLV vector [3], an HIV vector [19], and an HIV vector packaged with an MLV integrase (HIVmIN) [19], in HeLa cells. The sequences were re-annotated according to the criteria indicated in Figure 1, and analyzed for their JASPAR TFBS motif content by Clover against appropriate pair-weighted backgrounds (Table S1). The box plots in Figure S2 show that MLV sequences are highly enriched in TFBSs compared to HIV sequences (83.9 vs. 29.1, Wilcoxon rank sum test, *p*<2.2e-16). Interestingly, the MLV integrase re-directs the integration of an HIV vector (HIVmIN) towards regions significantly enriched in TFBSs (Figure S2, Wilcoxon rank sum test, *p*<2.2e-16; complete statistics in Table S2), independently from the intergenic (grey), intragenic (green) or TSS-proximal (yellow) location of the integration site (Figure 8A). Analysis of evolutionarily conserved TFBSs indicated a similar, statistically significant trend (Figure S3). As expected, the CpG island content increased significantly around the HIVmIN vector integration sites (4.9 vs. 0.2% in HIV sequences) (Figure 1).

A two-way hierarchical cluster analysis shows that MLV and HIV sequences are defined by substantially different patterns of over-represented motifs. Both the row (right) and the bootstrapped (top) dendrograms clearly separate MLV from HIV sequences. Most importantly, HIVmIN sequences are associated to MLV sequences in the bootstrapped dendrogram, and share most of their characteristic TFBS motifs with MLV sequences. These include a 7-motif branch (MA0099, MA0003, MA0063, MA0021, MA0026, MA0084, and MA0012) that is significantly under-represented in HIV sequences in the column dendrogram (Figures 8B and S1).

**Figure 9:**
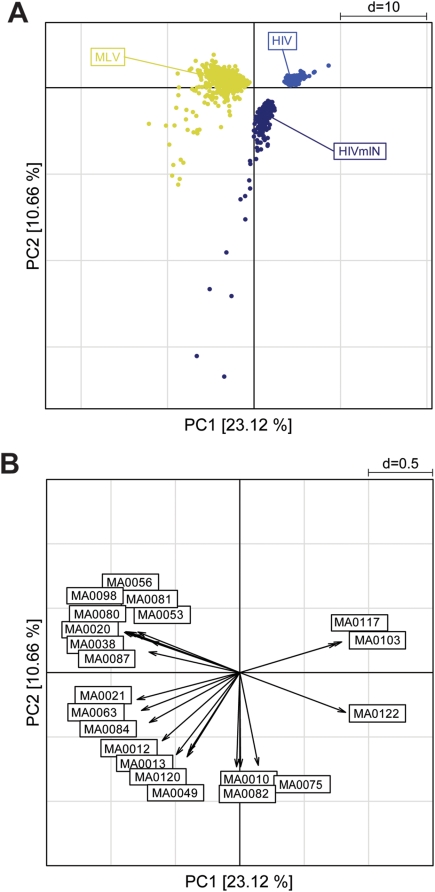
Principal component analysis of likelihood ratio values from the Clover analysis of the 49 JASPAR motifs enriched around integration sites of an MLV vector, an HIV vector and an HIV vector packaged with an MLV integrase (HIVmIN) in HeLa cells. (A) The scatter plot of the first two PCs (assessing 33.78% of total variability) reveals three main groups, corresponding to the vector type. The first component, accounting for 23.12% of the total variability, discriminates MLV from HIV sequences. The second component discriminates HIV from HIVmIN sequences but does not distinguish MLV from HIVmIN sequences. (B) The corresponding loading plot shows a set of MLV-specific motifs (MA0056, MA0098, MA0081, MA0080, MA0053, MA0020, MA0038 and MA0087), and a second group of motifs in common between HIVmIN and MLV sequences (MA0084, MA0063, MA0021, MA0012, MA0120, MA0013 and MA0049). All motifs are identified in Figure 5.

A PCA (Figure 9) confirmed the cluster analysis. The scatter plot of the first two components (accounting for 33.78% of the total variability) reveals three main groups, corresponding to the vector type. The first component, accounting for 23.12% of the total variability, discriminates the MLV from the HIV sequences. The second component discriminates HIV from HIVmIN sequences but does not distinguish MLV from HIVmIN sequences (Figure 9A). The corresponding loading plot (Figure 9B) shows a peculiar set of 8 motifs associated with MLV sequences, mostly belonging to the ETS family (MA0056, MA0098, MA0081, MA0080, MA0053, MA0020, MA0038, MA0087). A second group of seven motifs, mostly belonging to the Zn-finger C_2_H_2_ family, is in common between HIVmIN and MLV sequences (MA0084, MA0063, MA0021, MA0012, MA0120, MA0013, and MA0049). Most of these motifs were identified also by the hierarchical cluster analysis (Figure 8B). All motifs identified in the loading plot are shown in Figure 5.

## Discussion

Retroviral vectors, like their parental viruses, have strong biases and preferences for their integration into the target cell genome, which differ significantly in different retroviral families. Gamma-retroviruses favor integrations near TSS and CpG islands, lentiviruses integrate preferentially within active transcription units, while alpha- and beta-retroviruses, such as the avian sarcoma-leukosis virus and the murine mammary tumor virus, appear to integrate randomly into their host cell genome [4], [37]. These alternative preferences have a significant impact in predicting the risk of insertional gene activation of retroviral gene transfer vectors. It has recently been suggested that HIV-derived LV vectors, due to their different integration preferences and LTR enhancer-free design, could be associated to a lower genotoxic risk compared to conventional RV vectors [14], [25], [38], [39]. However, the current poor understanding of the molecular mechanisms underlying retroviral target site selection is a serious obstacle in the rational design of safer and more efficient gene transfer technology. Understanding in more detail the interactions between retroviral PICs and the human genome, the viral and cellular determinants of target site selection, and the role of the functional vector components (enhancers, promoters, splicing and polyadenylation signals) in influencing integration and gene expression, is crucial to assess the genotoxic characteristics of different vector families and designs.

Our study identifies a previously unrecognized feature of the regions targeted by RV PICs, i.e., an elevated content of transcription factor binding sites. By analyzing the sequences flanking the integration sites of MLV- and HIV-derived vectors in human HSCs, and of mutants featuring deletions and replacements of the LTR U3 enhancers, we show that integration in TFBS-rich regions of the genome is a specific characteristic of an RV vector with an LTR containing an RV enhancer (either MLV- or SFFV-derived). Deletion of the U3 element eliminates the TFBS over-representation around the insertion sites and reduces the relative frequency of TSS-proximal integrations, indicating that the U3 enhancer is an important viral determinant of RV target site selection. A statistical analysis indicates that over-representation of TFBSs is independent from the relative position or distance of the integration sites with respect to transcription units. This suggests that selection of TFBS-rich regions may in fact underlie all known RV integration preferences, particularly that for TSSs, CpG islands and DNase-I hypersensitive sites [3], [4], [17]–[19], [21], [27], where TFBS-rich regulatory regions are highly represented.

On the contrary, TFBS motifs are significantly under-represented around LV integration sites, independently from the presence of the HIV U3 element in the LTR. Replacement of the HIV with an MLV U3 element in an LV vector removes this negative bias, but is not sufficient to introduce a positive one like that of MLV-based vectors. Interestingly, when we analyzed the sequences around a previously published collection of integration sites of MLV, HIV, and an HIV vector packaged with an MLV integrase (HIVmIN) in HeLa cells [19], we discovered that the MLV integrase re-directs the integration of an HIV vector towards regions significantly enriched in TFBSs, again independently from intergenic, intragenic or TSS-proximal annotation of the integration site. Increased targeting of TFBS-rich regions might in turn underlie the increased targeting of TSSs, CpG islands and DNase-I hypersensitive sites previously observed for the HIVmIN vector [19]. We conclude that the MLV integrase and the LTR U3 region are the major viral determinants of the RV-specific selection of TFBS-rich target sites into the genome.

The MLV U3 enhancer contains repeated binding sites for a number of TFs, including members of the ETS, NFAT, C/EBP and nuclear hormone receptor families, the AML1/RUNX1-CBFB complex, and YY1. Bound transcription factors may very well be the cellular mediators of the LTR-associated component of RV integration preferences. Indeed, unintegrated retroviral genomes, possibly including PICs, are transcriptionally active in the target cell nuclei [40], [41], and are therefore likely to bind at least some of the TFs driving transcription of the integrated proviral genomes. We propose that TFs binding the U3 enhancer cooperate with the integrase in directing PICs towards regulatory regions actively engaged by the transcriptional machinery. Alternatively, unbound U3 elements in RV PICs engage chromatin-associated TFs to direct integration. Cooperation between TFs and the integrase may be seen as an evolution of the mechanisms by which yeast retrotransposons target their integration to specific genomic regions through tethering to host cell proteins. In *Saccharomyces cerevisiae*, a specific domain of the retrotransposase directs integration of the Ty3 retrotransposon to promoters transcribed by the DNA polymerase III, by tethering to Pol III-specific transcription factors (reviewed in [5]). This domain is lacking in the RV integrases, which are otherwise related to retrotransposases, and may have been functionally replaced by the association with TFs bound to the LTR elements. As a result, RV PICs are able to target a large collection of Pol II-specific, rather than a few Pol III-specific, regulatory elements throughout the genome.

A comparison between the TFBS motifs associated to RV integration sites in HSCs and in the non-hematopoietic HeLa cells shows a statistically significant association of specific motif patterns to either cell type, together with motifs over-represented in both cells. The existence of cell-specific and non-specific TFBS clusters suggests that RV PICs interact with general components of the enhancer-binding complexes (e.g., co-regulators, chromatin remodeling or mediator complexes) rather than with specific TFs or TF families. Recent data indicate that the MLV integrase may interact directly with chromatin-remodeling, DNA repair and transcription factors [42], providing independent, biochemical support to this hypothesis. Tethering of PICs to transcription factories, where promoters and regulatory regions are relocated by cell-specific mechanisms, may in turn be the cause of the RV-specific, high frequency of integration hot spots and preferred targeting of genes associated to cell-specific regulatory networks [21], [27]. Indeed, TFBSs specifically associated with RV integration in HSCs include binding sites for HSC-specific regulators of cell proliferation, differentiation or quiescence, such as LMO2, AML1/RUNX1, and FOXO3. A mechanism coupling target site selection to gene regulation may have evolved to maximize the probability for gamma-retroviruses to be transcribed in the target cell genome, and possibly to induce expansion of infected cells by insertional activation of cell-specific growth regulators. Interestingly, integration of HIV does not favor, and possibly avoids, TFBS-rich regions, suggesting the evolution of a different mechanism that targets open chromatin regions while minimizing interference with the cell transcriptional machinery. Recent data emerging from large integration site datasets predict the association of HIV integration sites with histone post-translational modifications specifically associated to transcribed chromatin rather than to enhancers, promoters and other regulatory regions [43]. Tethering via the LEDGF/p75 chromatin component is likely to play a major role in mediating this targeting strategy [12], [44], [45].

The different propensity of RV and LV vectors to target regulatory regions has an obvious impact on the design of gene transfer vectors for clinical applications. Although a self-inactivating (ΔU3) design is predicted, also by this analysis, to improve the safety characteristics of MLV-based vectors, the activity of the MLV integrase remains an undesirable enhancer of the frequency by which an RV vector may target potentially dangerous regions of the genome. This study also shows the importance of the cell context in determining the frequency of integration into certain genomic regions, and predicts that targeting of dominantly acting proto-oncogenes may have a different likelihood in different cells. As an example, the LMO2 locus is targeted at very high frequency in HSCs [22], [27] but not in T-cells where it is not expressed (unpublished observation). On the contrary, the use of HIV-based vectors would minimize insertional gene activation by generally reducing integration in the proximity of active promoters and enhancers. Analysis of TFBSs close to the integration sites provides an additional readout to study the potential genotoxicity of vectors containing different promoters, enhancers and regulatory elements in a specific cell context.

## Materials and Methods

### Retroviral vectors

MLV-derived oncoretroviral vectors containing a green fluorescent protein (GFP) gene, an adenosine deaminase (ADA) or a γ_c_ receptor cDNA under the control of a wild-type MLV LTRs were the previously described LGSΔN [46], GIADA [47] and MFG-γ_c_ [48] vectors, respectively. The ΔU3-MLV vector carried a GFP gene under the control of a ΔU3 (−413 to −62) LTR, and was previously described as LGSΔN-ΔCAAT [46]. The SFFV-MLV vector expressed the GFP gene under the control of the SFFV LTR in the previously described pSF91 MLV vector backbone [49] (a gift from C. Baum, Hanover). HIV vectors with wild-type LTRs were the previously described pHR2pptCMV-GFPwpre and pHR2pptGSΔN LV vectors [27], in which the HIV-1 wild-type LTR drove the expression of the GFP gene. The ΔU3-HIV[CMV] vector carried −418 to −18 deletion in the U3 region and an internal GFP expression cassette driven by the cytomegalovirus (CMV) immediate-early promoter, and was previously described as pRRLsin-18.pptCMV-GFPwpre [50]. The ΔU3-HIV[MLV] vector carried a −418 to −40 U3 deletion and was obtained by inserting an internal ΔLNGFR expression cassette driven by the full MLV LTR into the pRRLsin-40.GFP vector [51]. The MLV-HIV vector was built by inserting the PCR-amplified −413 to −62 fragment of the MLV U3 region at position −40 in the HIV LTR of the pRRLsin-40.GFP vector [51], and adding an internal SV40-driven ΔLNGFR expression cassette.

RV vector supernatants were produced by transient transfection of the amphotropic Phoenix packaging cell line. Infectious particle titer was determined on the K562 human hematopoietic cell line. The pSF91.eGFP.WPRE RV vector was VSV-G pseudotyped by transient co-transfection of 293T cells with an MLV *gag/pol* expression plasmid (a gift from C. Baum) and a VSV-G expression plasmid. Infectious particle titer was determined on 293T cells. VSV-G pseudotyped LV particles were prepared by transient co-transfection of 293T cells, collected and concentrated as described [52], and titrated on 293T cells. Transduction efficiency was evaluated by scoring GFP and/or ΔLNGFR transgene expression on target cells by flow cytometry. The ADA and γ_c_ receptor RV vectors were produced as amphotropic or GaLV envelope-pseudotyped particles from stable packaging cell lines, and titrated as previously described [47], [48].

### Transduction of target cells

Human CD34^+^ HSCs were purified from the Ficoll fraction of donor cord blood, bone marrow or peripheral blood by the CD34 magnetic cell isolation kit (MiniMACS; Miltenyi, Auburn, CA) and pre-stimulated for 24–48 hours at a density of 1×10^6^ cells/ml in serum-free Iscove's Modified Dulbecco's Medium (IMDM; BioWhittaker; Verviers, Belgium), supplemented with 20% BIT serum substitute, recombinant human thrombopoietin, interleukin-3, stem cell factor and Flt-3 ligand, as previously described [27]. Transduction with RV vectors was performed by spinoculation (3 rounds at 1,500 rpm for 45 min) in the presence of retroviral supernatants and 4 µg/ml polybrene. Transduction with LV vectors was performed by over-night incubation of CD34^+^ cells with vector stocks at a multiplicity of infection (MOI) of 200 in the presence of 4 µg/ml polybrene. Transduction efficiency was evaluated by flow cytometry as described [27]. Transduced cells were collected 5–12 days after infection and phenotyped with a RPE-conjugated anti-human CD34 antibody (Becton Dickinson) before DNA extraction.

SupT1 cells were grown in RPMI 1640 (BioWhittaker) supplemented with 10% fetal bovine serum, and transduced with MLV-HIV viral stocks at an MOI of 25 in the presence of 8 µg/ml polybrene. After virus addition, cells were spinoculated for 1 hour (1,800 rpm, 4°C) and left at 4°C for another hour to ensure a synchronous infection. Cells were then transferred to a 37°C incubator and cross-linked after 10 hours for chromatin immunoprecipitation (ChIP) analysis on pre-integration complexes, or left in culture for 2 additional weeks for ChIP analysis on integrated proviruses.

### Sequencing, mapping and annotation of retroviral integration sites

Integration sites were cloned by linker-mediated PCR (LM-PCR) as described [3], [53]. Briefly, genomic DNA was extracted from 0.5–5×10^6^ infected cells and digested with *Mse*I and a second enzyme to prevent amplification of internal 5′ LTR fragments (*Pst*I for RV vectors and *Sac*I/*Nar*I for LV vectors). An *Mse*I double-stranded linker was then ligated and LM-PCR performed with nested primers specific for the linker and the 3′ LTR (MLV: 5′- GACTTGTGGTCTCGCTGTTCCTTGG-3′ and 5′- GGTCTCCTCTGAGTGATTGACTACC-3′; HIV: 5′- AGTGCTTCAAGTAGTGTGTGCC-3′ and 5′- GTCTGTTGTGTGACTCTGGTAAC-3′). PCR products were shotgun-cloned (TOPO TA cloning kit, Invitrogen; Carlsbad, CA) into libraries of integration junctions, which were then sequenced to saturation. A valid integration contained the MLV or HIV nested primer, the entire MLV or HIV genome up to a CA dinucleotide and the linker nested primer. Sequences between the 3′ LTR and the linker primers were mapped onto the human genome (UCSC Human Genome Project Working Draft, hg17) using Blat[54] requiring a 98% identity over the entire sequence length and selecting the best hit. The absolute genomic coordinates of the integration sites where defined as a result of the combination of genomic alignment and vector relative orientation data. Random genomic sequences originated by LM-PCR (genomic *Mse*I-*Mse*I, *Pst*I-*Mse*I, *Nar*I-*Mse*I or *Sac*I-*Mse*I fragments) were mapped by the same criteria, and used as experimental controls. All sequences were annotated as “TSS-proximal” when occurring at a distance of ±5 kb from the TSS of any Known Gene (UCSC definition), “intragenic” when occurring within the transcribed portion of at least one Known Gene >5 kb from the TSS, and “intergenic” in all other cases. Whenever multiple transcript variants exist, the most represented and/or the longest isoform was chosen. Integration sites from published datasets [3], [19] were re-mapped and annotated according to the same criteria.

### Analysis of transcription factor binding sites

TFBS analysis was carried out on genomic sequences encompassing each integration site with ±1.0 kb of sequence length. Based on the TSS-proximal/intragenic/intergenic annotation of each integration site, we grouped datasets that do not significantly differ from each other (two-sided test on equal proportion) into seven groups of integration preferences, and generated the same number of random weighted control groups of sequences that reproduce, in proportion, the specific integration preference of each vector. Each fitted background was composed of 10,000 sequences of 2.0 kb in length derived from 100,000 randomly generated integration sites throughout the genome (Table S1). TFBS enrichment analysis was carried out with the Clover program [34], with dinucleotide randomization. Motif *p*-value threshold was set to 0.05. TFBSs, described as positional-weight matrices, were obtained from the JASPAR Core 2005 database of experimentally validated motifs [35]. Each sequence set was paired with the appropriate weighted background. TFBSs having a global *p*-value<0.05 were considered as significantly enriched in the test sequences and selected for analysis. Motif frequency was defined as the number of motif per sequence significantly enriched in the Clover analysis, applying a one-sided Wilcoxon rank sum test (alternative hypothesis: “greater”). Motif likelihood ratios, obtained by Clover, were used for cluster analysis and PCA. Analysis of conserved TFBSs was performed using the TFBS Conserved Track at UCSC Genome Browser, which includes binding sites conserved between the human and mouse or rat genome alignment (188 human matrices from the TRANSFAC Matrix Database v 7.0). After determination of the total count of matrices that match in each 2.0-kb test sequence, random and matched fitted backgrounds, a Fisher exact test (two-sided, confidence level = 0.95) was used to determine statistical significance. The STAMP tool-kit [55] was used to match JASPAR and TRANSFAC matrices using default parameters.

For the hierarchical clustering analysis, data were scaled on motifs columns before analysis. Column dendrograms were sampled with 10,000 bootstrap replicates [36]. Nodes having an Approximately Unbiased (AU) *p*-value>0.95 were scored as significant and stable nodes. PCA was computed on correlation matrix without factor rotation. For each bidimensional plane considered, only loadings having a value higher than cos(π/4)∼0.707 were considered as relevant: since all vectors have a length = 1 in poly-dimensional space, we only see their projection on the principal components plane, hence if the projection length is longer than 0.707 the angle between the vector and the plane is less than π/4 (45 degrees), meaning strong correlation between the vector and the plane.

All statistical analyses were performed using the R language and environment for statistical computing and graphics version 2.6.2 (http://www.R-project.org) and several contributed packages. Hierarchical clustering used the *pvclust* package; PCA alysis used *ade4*; parallel processing was implemented using the *snow* package. Stats package was used for the others analysis.

## Supporting Information

Figure S1 (0.16 MB PDF)

Figure S2 (0.11 MB PDF)

Figure S3 (0.05 MB PDF)

Table S1 (0.05 MB PDF)

Table S2 (0.05 MB PDF)

Table S3 (0.08 MB PDF)

Table S4 (0.05 MB PDF)

Table S5 (0.04 MB PDF)
