# Supplementary material for: Transcription Factor Binding Sites Are Genetic Determinants of Retroviral Integration in the Human Genome
Source: PLoS One. 2009 Feb 24;4(2):e4571. doi: 10.1371/journal.pone.0004571 (PMC2642719; doi:10.1371/journal.pone.0004571)
Supplement: Figure S1 — (0.16 MB PDF) [file pone.0004571.s001.pdf]

a

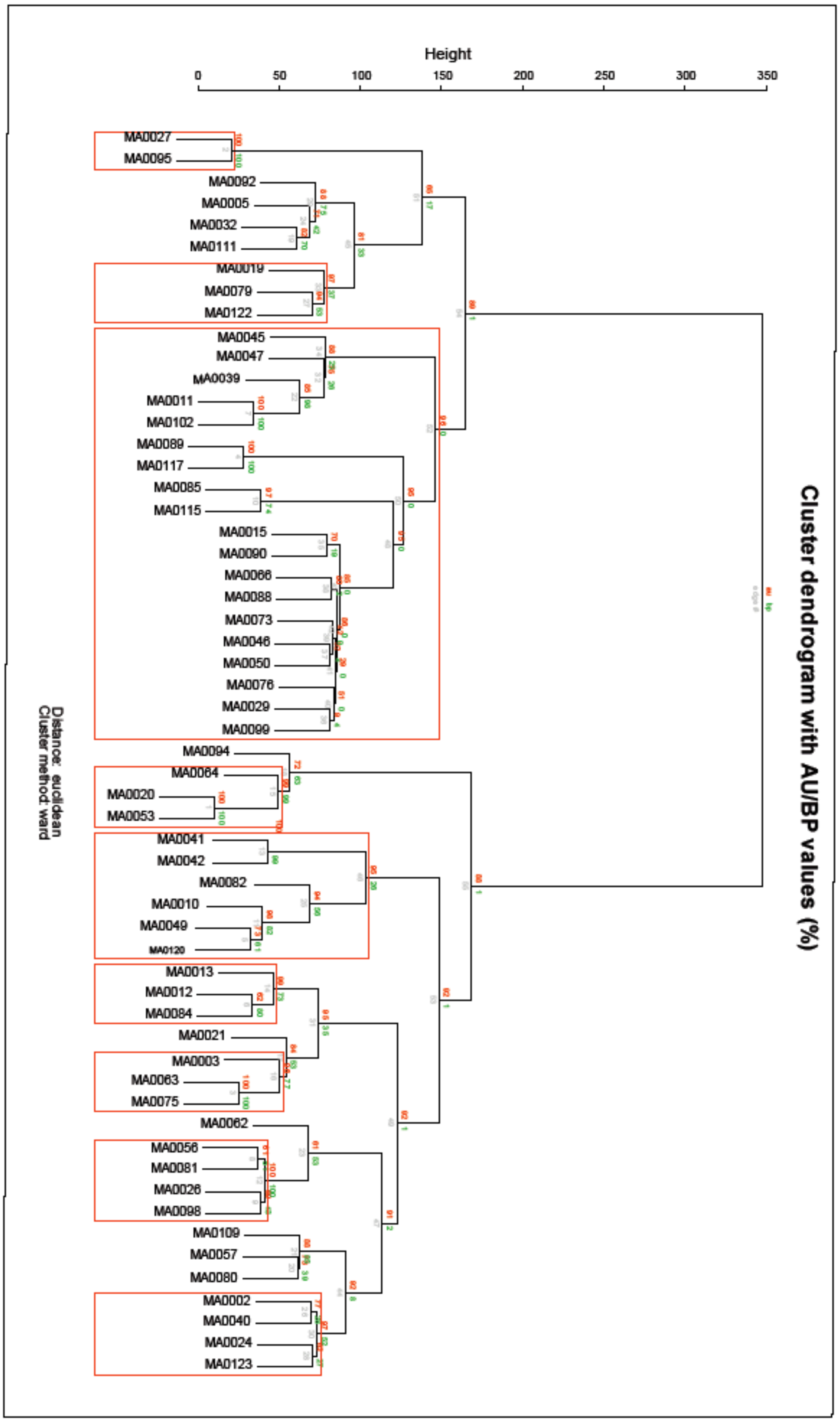

Cluster dendrogram with AU/BP values (%)
Height
0
50
100
150
200
250
300
350
MA0027
MA0095
MA0092
MA0005
MA0032
MA0111
MA0019
MA0079
MA0122
MA0045
MA0047
MA0039
MA0011
MA0102
MA0089
MA0117
MA0085
MA0115
MA0015
MA0090
MA0066
MA0088
MA0073
MA0046
MA0050
MA0076
MA0029
MA0099
MA0094
MA0064
MA0020
MA0053
MA0041
MA0042
MA0082
MA0010
MA0049
MA0120
MA0013
MA0012
MA0084
MA0021
MA0003
MA0063
MA0075
MA0062
MA0056
MA0081
MA0026
MA0098
MA0109
MA0057
MA0080
MA0002
MA0040
MA0024
MA0123
Distance: euclidean
Cluster method: ward

b

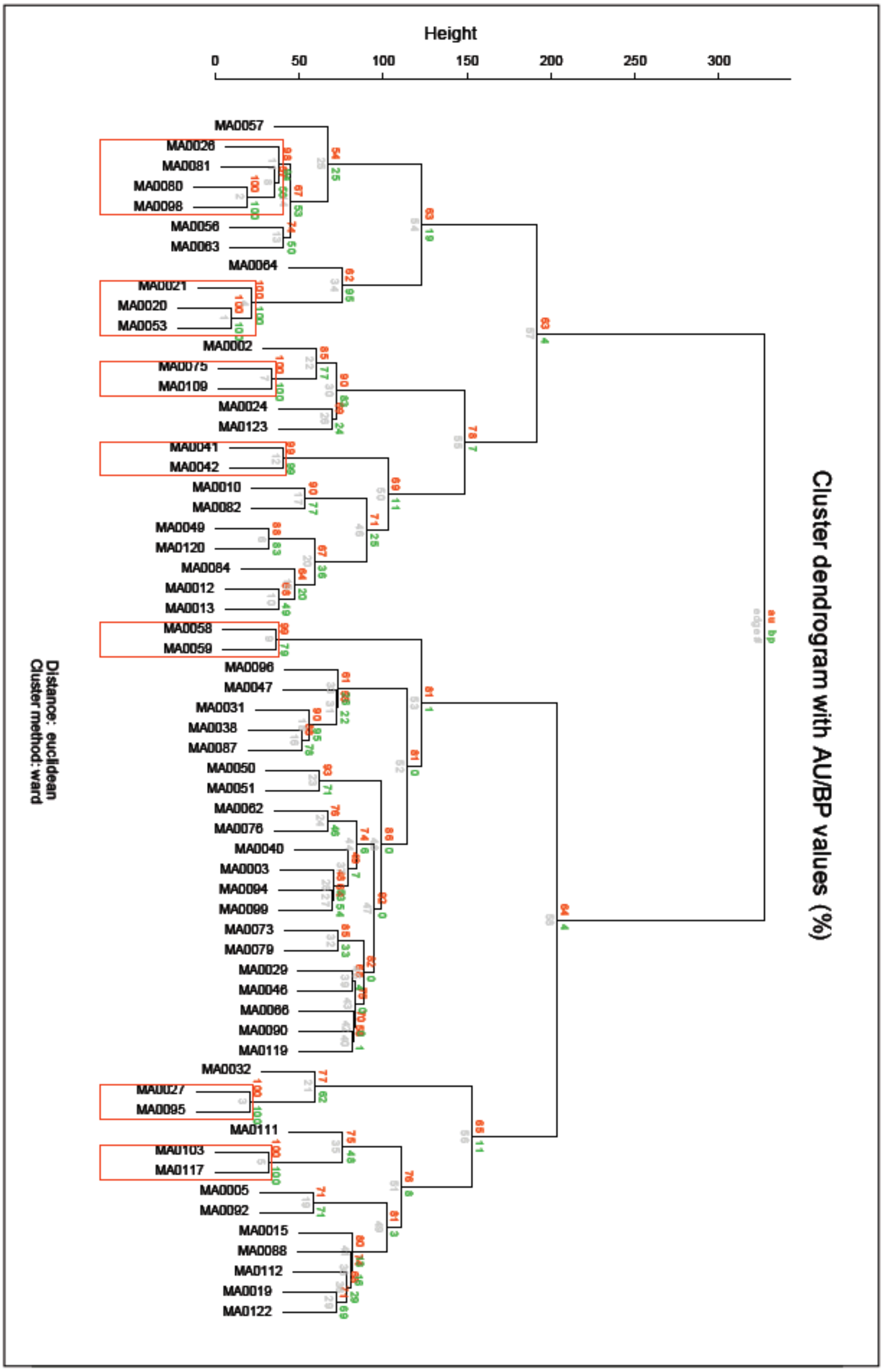

Height
0
50
100
150
200
250
300
MA0057
MA0026
MA0081
MA0080
MA0098
MA0056
MA0063
MA0064
MA0021
MA0020
MA0053
MA0002
MA0075
MA0109
MA0024
MA0123
MA0041
MA0042
MA0010
MA0082
MA0049
MA0120
MA0084
MA0012
MA0013
MA0058
MA0059
MA0096
MA0047
MA0031
MA0038
MA0087
MA0050
MA0051
MA0062
MA0076
MA0040
MA0003
MA0094
MA0099
MA0073
MA0079
MA0029
MA0046
MA0066
MA0090
MA0119
MA0032
MA0027
MA0095
MA0111
MA0103
MA0117
MA0005
MA0092
MA0015
MA0088
MA0112
MA0019
MA0122
Distance: euclidean
Cluster method: ward
Cluster dendrogram with AU/BP values (%)
au bp
edge #

c

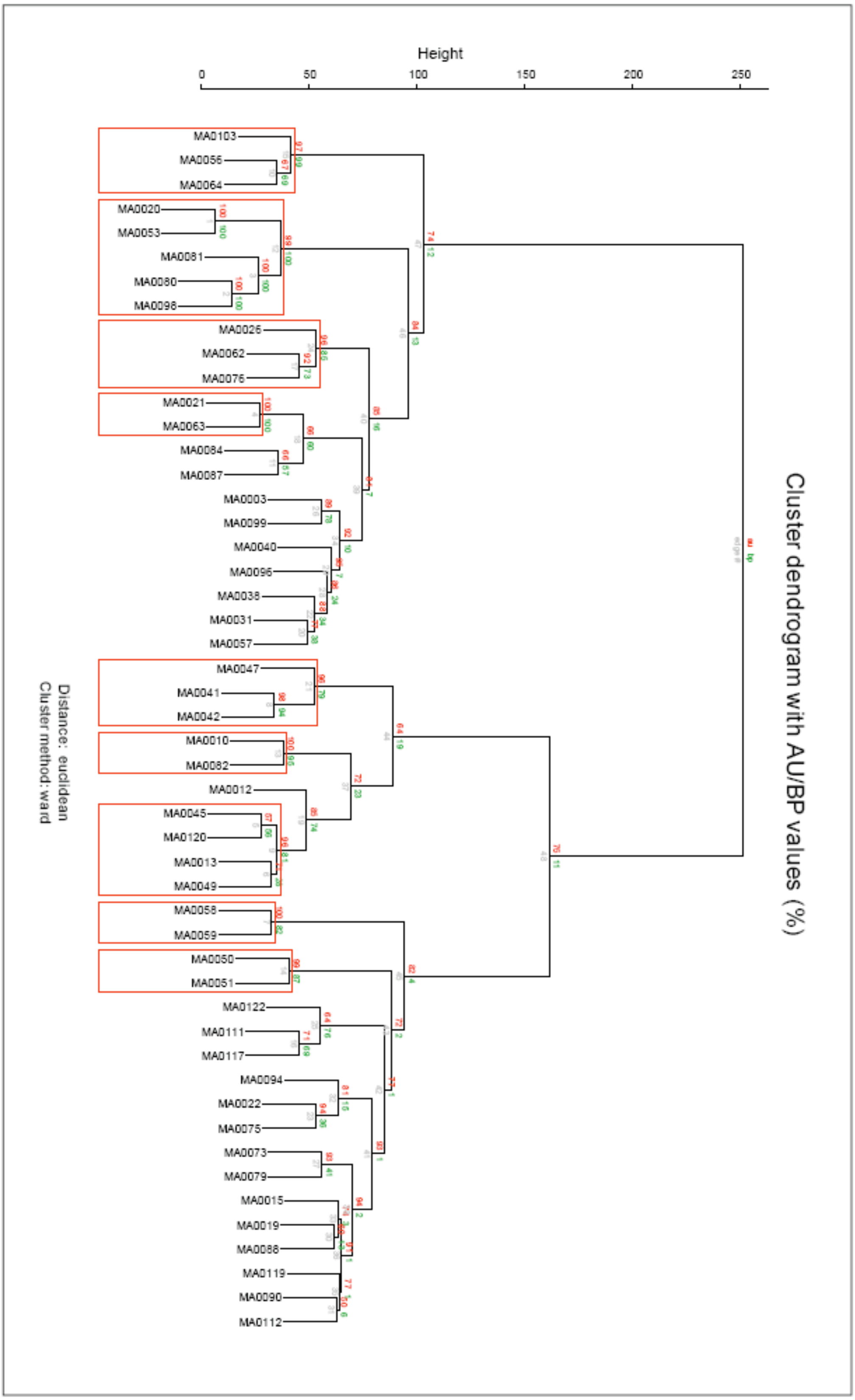

Cluster dendrogram with AU/BP values (%)
Height
0
50
100
150
200
250
MA0103
MA0056
MA0064
MA0020
MA0053
MA0081
MA0080
MA0098
MA0026
MA0062
MA0076
MA0021
MA0063
MA0084
MA0087
MA0003
MA0099
MA0040
MA0096
MA0038
MA0031
MA0057
MA0047
MA0041
MA0042
MA0010
MA0082
MA0012
MA0045
MA0120
MA0013
MA0049
MA0058
MA0059
MA0050
MA0051
MA0122
MA0111
MA0117
MA0094
MA0022
MA0075
MA0073
MA0079
MA0015
MA0019
MA0086
MA0119
MA0090
MA0112
au
bp
edge #
Distance: euclidean
Cluster method: ward

Figure S1. Bootstrapped matrix column dendrograms of the hierarchical cluster analysis shown in Figures 3, 7A and 8B. Column dendrograms have been sampled with 10,000 bootstrap replicates and approximately unbiased probabilities are reported on each node (AU, red). Red rectangles on the tree identify nodes with an AU value >0.95, hence considered significant, stable nodes. **a)** Bootstrapped matrix column dendrograms of the hierarchical cluster analysis shown in Figures 3 (RV and LV vectors in $CD34^+$ HSCs). **b)** Bootstrapped matrix column dendrograms of the hierarchical cluster analysis shown in Figures 7A (MLV and HIV vectors in $CD34^+$ HSCs and HeLa cells. **c)** Bootstrapped matrix column dendrograms of the hierarchical cluster analysis shown in Figures 8B (HIV, MLV and HIVmIN vectors in HeLa cells).
