# Supplementary material for: Transcription Factor Binding Sites Are Genetic Determinants of Retroviral Integration in the Human Genome
Source: PLoS One. 2009 Feb 24;4(2):e4571. doi: 10.1371/journal.pone.0004571 (PMC2642719; doi:10.1371/journal.pone.0004571)
Supplement: Figure S2 — (0.11 MB PDF) [file pone.0004571.s002.pdf]

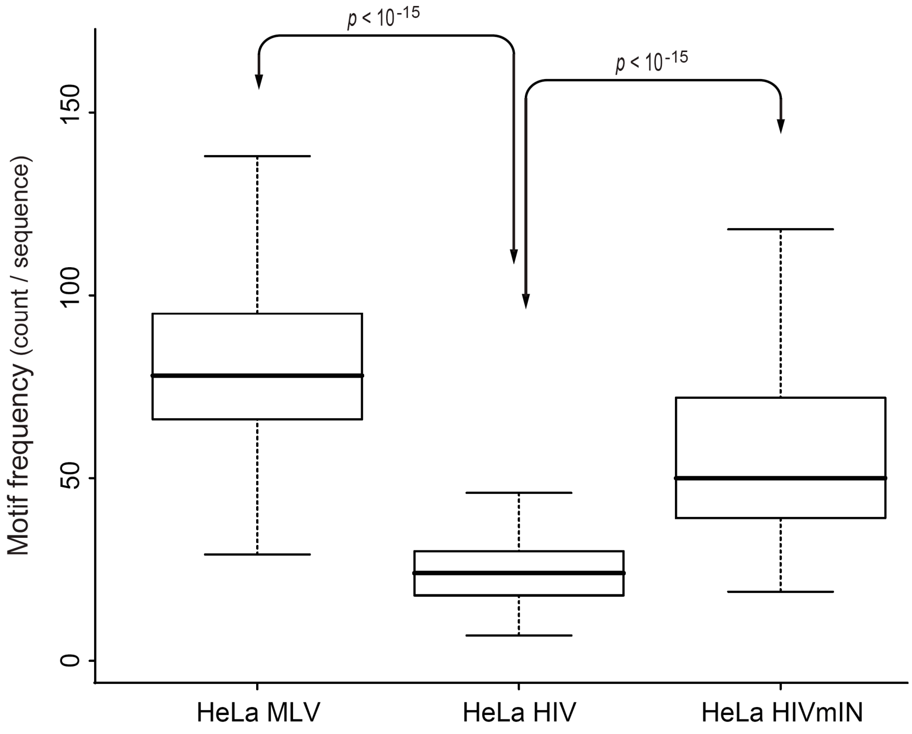

Figure S2. Box plot of the frequency of TFBSs (number of enriched motifs per sequence) around integration sites in HeLa cells of an MLV vector, an HIV vector, and an HIV vector packaged with an MLV integrase (HIVmIN) (vectors are identified in Figure 1). Statistical significance of differences in TFBS frequencies among groups (Wilcoxon rank sum test) is reported in Table S2. The *p*-value of some significant comparisons is indicated.
