# Supplementary material for: Transcription Factor Binding Sites Are Genetic Determinants of Retroviral Integration in the Human Genome
Source: PLoS One. 2009 Feb 24;4(2):e4571. doi: 10.1371/journal.pone.0004571 (PMC2642719; doi:10.1371/journal.pone.0004571)
Supplement: Figure S3 — (0.05 MB PDF) [file pone.0004571.s003.pdf]

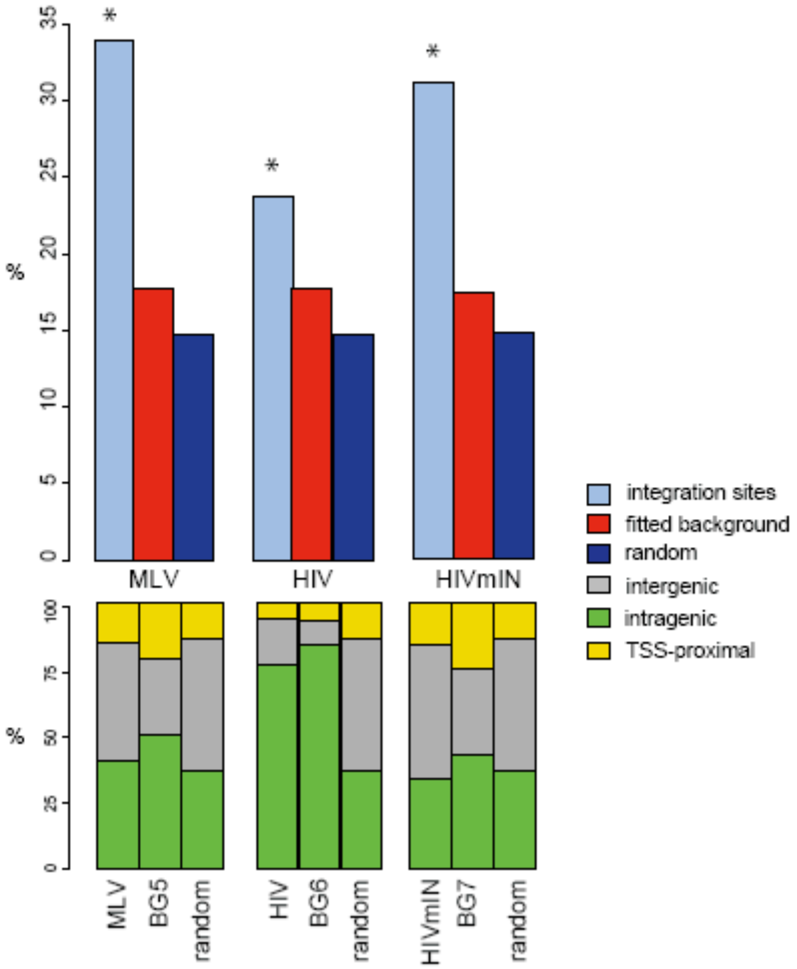

Figure S3. Analysis of the frequency of evolutionarily conserved TFBSs in genomic sequences flanking integration sites of an MLV vector, an HIV vector and an HIV vector packaged with an MLV integrase (HIVmIN) in HeLa cells, using 188 matrices conserved in a human-mouse and/or –rat alignment (HMR Conserved Transcription Factor Binding Sites table at UCSC) as a motif database. In the upper panel, data are plotted as percentage of sequences containing at least one conserved TFBS. Each group of sequences (light blue bars) is compared to a weighted (red bars) and a random (blue bars) computational control sequence set (see methods for definitions). Asterisks highlight experimental groups that show a significant enrichment of frequency compared to control sets (one-sided Fisher test, complete statistics in Table S4). In the lower panel, all frequency data are broken down in three subgroups according to the integration site annotation, i.e., intergenic (grey bars), TSS-proximal (yellow bars) and intragenic (green bars). The complete list of conserved motifs and their distribution over the different datasets are reported in Table S5.
