# Supplementary material for: Transcription Factor Binding Sites Are Genetic Determinants of Retroviral Integration in the Human Genome
Source: PLoS One. 2009 Feb 24;4(2):e4571. doi: 10.1371/journal.pone.0004571 (PMC2642719; doi:10.1371/journal.pone.0004571)
Supplement: Table S1 — (0.05 MB PDF) [file pone.0004571.s004.pdf]

Supplementary Table 1

Background definition and assignment of the corresponding experimental groups. We randomly generated seven weighted control groups of sequences that reproduce, in proportion, the integration preferences of each vector set; these were then used as pair-weighted background for TFBS analysis.

Fitted random groups

Background groups	Intergenic %	TSS-prox %	Intragenic %	Corresponding experimental group
BG1	59.8	4.5	35.7	Controls
BG2	37.0	16.5	46.4	MLV(CD34$^+$)
BG3	28.0	8.4	63.5	HIV(CD34$^+$), ΔU3-HIV[CMV], ΔU3-HIV[MLV], MLV-HIV
BG4	41.6	19.9	38.5	SFFV-MLV
BG5	46.3	13.4	40.3	MLV(Hela), ΔU3-MLV
BG6	17.3	5.6	77.1	HIV(Hela)
BG7	50.8	15.7	33.5	HIVmIN
