# Supplementary material for: Transcription Factor Binding Sites Are Genetic Determinants of Retroviral Integration in the Human Genome
Source: PLoS One. 2009 Feb 24;4(2):e4571. doi: 10.1371/journal.pone.0004571 (PMC2642719; doi:10.1371/journal.pone.0004571)
Supplement: Table S2 — (0.05 MB PDF) [file pone.0004571.s005.pdf]

**Supplementary Table 2**

Comparison of frequency of TFBS distributions (number of enriched motifs per sequence). All combinations of sample diversities were in turn assessed by exact Wilcoxon rank sum test (one-sided, alternative hypothesis: "greater"). The control group distribution is significantly shifted to the right (greater) with respect to HIV, ΔU3-HIV[CMV] and ΔU3-HIV[MLV] (p-value <2.2e-16), but not to MLV, ΔU3-MLV, SFFV-MLV and MLV-HIV (p-value =1). The MLV distribution is instead significantly shifted to the right (greater) of all the other distributions, exception mande for the SFFV-MLV distribution group. An asterisk specifies the values highlighted in Figure 2A.

***CD34$^+$ HSC cells***

| | Controls | MLV | ΔU3-MLV | SFFV-MLV | HIV | ΔU3-HIV[CMV] | ΔU3-HIV[MLV] | MLV-HIV |
|---|---|---|---|---|---|---|---|---|
| **Controls** | - | 1 | 1 | 1 | <2.2e-16 | <2.2e-16 | <2.2e-16 | 1 |
| **MLV** | <2.2e-16 (*) | - | <2.2e-16 (*) | 0,900 | <2.2e-16 | <2.2e-16 | <2.2e-16 | <2.2e-16 |
| **ΔU3-MLV** | <2.2e-16 | 1 | - | 1 | <2.2e-16 | <2.2e-16 | <2.2e-16 | 4.4e-16 |
| **SFFV-MLV** | <2.2e-16 | 0,100 | <2.2e-16 | - | <2.2e-16 | <2.2e-16 | <2.2e-16 | <2.2e-16 |
| **HIV** | 1 | 1 | 1 | 1 | - | 1 | 1.6e-08 | 1 |
| **ΔU3-HIV[CMV]** | 1 | 1 | 1 | 1 | 6.6e-13 | - | <2.2e-16 | 1 |
| **ΔU3-HIV[MLV]** | 1 | 1 | 1 | 1 | 1 | 1 | - | 1 |
| **MLV-HIV** | 1.4e-08 | 1 | 1 | 1 | <2.2e-16 | <2.2e-16 | <2.2e-16 (*) | - |

***Hela cells***

| | MLV | HIV | HIVmIN |
|---|---|---|---|
| **MLV** | - | <2.2e-16 | <2.2e-16 |
| **HIV** | 1 | - | 1 |
| **HIVmIN** | 1 | <2.2e-16 | - |
