# Supplementary material for: Transcription Factor Binding Sites Are Genetic Determinants of Retroviral Integration in the Human Genome
Source: PLoS One. 2009 Feb 24;4(2):e4571. doi: 10.1371/journal.pone.0004571 (PMC2642719; doi:10.1371/journal.pone.0004571)
Supplement: Table S3 — (0.08 MB PDF) [file pone.0004571.s006.pdf]

Supplementary Table 3

Distribution of JASPAR motifs (average number of motifs found enriched per sequence and 1st to 99th percentile range) in sequences flanking (+/- 1,000 bp) the integration sites of different MLV and HIV vectors in human HSCs (see Figure 1 for vector identification).

Matrix ID	TF name	TF class	Controls		MLV		ΔU3-MLV		SFFV-MLV		HIV		ΔU3-HIV[CMV]		ΔU3-HIV[MLV]		MLV-HIV	
			mean	1-99th percentile	mean	1-99th percentile	mean	1-99th percentile	mean	1-99th percentile	mean	1-99th percentile	mean	1-99th percentile	mean	1-99th percentile	mean	1-99th percentile
MA0001	AGL3	MADS	0	0	0	0	0	0	0	0	0	0	0	0	0	0	0	0
MA0002	RUNX1	RUNT	0	0-0	1.38	0-4	0	0-0	0	0-0	0	0-0	0	0-0	0	0-0	0	0-0
MA0003	TFAP2A	AP2	0	0-0	3.28	0-11	0	0-0	3.27	0-10	0	0-0	0	0-0	0	0-0	0	0-0
MA0004	Arnt	bHLH	0	0	0	0	0	0	0	0	0	0	0	0	0	0	0	0
MA0005	Agamous	MADS	0	0-0	0	0-0	0	0-0	0	0-0	0	0-0	1.64	0-6	0	0-0	1.55	0-5
MA0006	Arnt-Ahr	bHLH	0	0	0	0	0	0	0	0	0	0	0	0	0	0	0	0
MA0007	Ar	NUCLEAR RECEPTOR	0	0	0	0	0	0	0	0	0	0	0	0	0	0	0	0
MA0008	Athb-1	HOMEO-ZIP	0	0	0	0	0	0	0	0	0	0	0	0	0	0	0	0
MA0009	T	T-BOX	0	0	0	0	0	0	0	0	0	0	0	0	0	0	0	0
MA0010	Broad-complex_1	ZN-FINGER, C2H2	0	0-0	4.53	0-28.72	0	0-0	0	0-0	0	0-0	0	0-0	0	0-0	0	0-0
MA0011	Broad-complex_2	ZN-FINGER, C2H2	0	0-0	0	0-0	0	0-0	1.12	0-5.96	0	0-0	0	0-0	0	0-0	0	0-0
MA0012	Broad-complex_3	ZN-FINGER, C2H2	0	0-0	1.84	0-11.72	0	0-0	2.11	0-10.18	0	0-0	0	0-0	0	0-0	0	0-0
MA0013	Broad-complex_4	ZN-FINGER, C2H2	0	0-0	2.77	0-20	0	0-0	3.00	0-24.12	0	0-0	0	0-0	0	0-0	0	0-0
MA0014	Pax5	PAIRED	0	0	0	0	0	0	0	0	0	0	0	0	0	0	0	0
MA0015	CF2-II	ZN-FINGER, C2H2	3.36	0-46	0	0-0	0	0-0	0	0-0	0	0-0	0	0-0	0	0-0	0	0-0
MA0016	CFI-USP	NUCLEAR RECEPTOR	0	0	0	0	0	0	0	0	0	0	0	0	0	0	0	0
MA0017	NR2F1	NUCLEAR RECEPTOR	0	0	0	0	0	0	0	0	0	0	0	0	0	0	0	0
MA0018	CREB1	bZIP	0	0	0	0	0	0	0	0	0	0	0	0	0	0	0	0
MA0019	Chop-cEBP	bZIP	0	0-0	0	0-0	0	0-0	0	0-0	2.63	0-9.98	2.70	0-10.56	3.19	0-10	0	0-0
MA0020	Dof2	ZN-FINGER, DOF	3.38	0-9	3.86	0-10	4.08	0-11.01	3.68	0-11.06	0	0-0	0	0-0	0	0-0	3.41	0-9.01
MA0021	Dof3	ZN-FINGER, DOF	0	0-0	3.50	0-10	0	0-0	3.77	0-11	0	0-0	0	0-0	0	0-0	2.91	0-7.01
MA0022	Dorsal_1	REL	0	0	0	0	0	0	0	0	0	0	0	0	0	0	0	0
MA0023	Dorsal_2	REL	0	0	0	0	0	0	0	0	0	0	0	0	0	0	0	0
MA0024	E2F1	Unknown	0	0-0	0.61	0-3	0	0-0	0	0-0	0	0-0	0	0-0	0	0-0	0	0-0
MA0025	NFIL3	bZIP	0	0	0	0	0	0	0	0	0	0	0	0	0	0	0	0
MA0026	E74A	ETS	0	0-0	2.34	0-7	2.37	0-6.01	2.30	0-8	0	0-0	0	0-0	0	0-0	0	0-0
MA0027	En1	HOMEO	0	0-0	0	0-0	0	0-0	0	0-0	0.64	0-3	0	0-0	0	0-0	0	0-0
MA0028	ELK1	ETS	0	0	0	0	0	0	0	0	0	0	0	0	0	0	0	0
MA0029	Evi1	ZN-FINGER, C2H2	0	0-0	0.83	0-4	0	0-0	0	0-0	0	0-0	0	0-0	0	0-0	0	0-0
MA0030	FOXF2	FORKHEAD	0	0	0	0	0	0	0	0	0	0	0	0	0	0	0	0
MA0031	FOXD1	FORKHEAD	0	0	0	0	0	0	0	0	0	0	0	0	0	0	0	0
MA0032	FOXC1	FORKHEAD	0	0-0	0	0-0	0	0-0	0	0-0	0.14	0-2	0.13	0-2	0.10	0-1.01	0.12	0-2
MA0032	FOXC1	FORKHEAD	0	0	0	0	0	0	0	0	0	0	0	0	0	0	0	0
MA0033	FOXL1	FORKHEAD	0	0	0	0	0	0	0	0	0	0	0	0	0	0	0	0
MA0034	GAMYB	TRP-CLUSTER	0	0	0	0	0	0	0	0	0	0	0	0	0	0	0	0
MA0035	Gata1	ZN-FINGER, GATA	0	0	0	0	0	0	0	0	0	0	0	0	0	0	0	0
MA0036	GATA2	ZN-FINGER, GATA	0	0	0	0	0	0	0	0	0	0	0	0	0	0	0	0
MA0037	GATA3	ZN-FINGER, GATA	0	0	0	0	0	0	0	0	0	0	0	0	0	0	0	0
MA0039	Klf4	ZN-FINGER, C2H2	0	0-0	0	0-0	4.98	0-13.02	4.97	0-13.12	0	0-0	0	0-0	0	0-0	0	0-0
MA0040	Foxq1	FORKHEAD	0	0-0	163,00	0-6	0	0-0	0	0-0	0	0-0	0	0-0	0	0-0	0	0-0

MA0041	Foxd3	FORKHEAD	0	0-0	4.18	0-22.72	0	0-0	4.70	0-26.06	0	0-0	0	0-0	0	0-0	0	0-0
MA0042	FOXI1	FORKHEAD	0	0-0	2.67	0-11	0	0-0	2.85	0-10.06	0	0-0	0	0-0	0	0-0	0	0-0
MA0043	HLF	bZIP	0	0	0	0	0	0	0	0	0	0	0	0	0	0	0	0
MA0044	HMG-1	HMG	0	0	0	0	0	0	0	0	0	0	0	0	0	0	0	0
MA0045	HMG-IY	HMG	0	0-0	0	0-0	0	0-0	6.85	0-31.06	0	0-0	0	0-0	0	0-0	0	0-0
MA0046	TCF1	HOMEO	0	0-0	1.05	0-5	0	0-0	1.23	0-5	0	0-0	0	0-0	0	0-0	0	0-0
MA0047	Foxa2	FORKHEAD	0	0-0	0	0-0	0	0-0	2.45	0-10.06	0	0-0	0	0-0	0	0-0	0	0-0
MA0048	NHLH1	bHLH	0	0	0	0	0	0	0	0	0	0	0	0	0	0	0	0
MA0049	Hunchback	ZN-FINGER, C2H2	0	0-0	6.31	0-32.72	0	0-0	7.13	0-37.36	0	0-0	0	0-0	0	0-0	0	0-0
MA0050	IRF1	TRP-CLUSTER	0	0-0	1.76	0-6	1.63	0-5	1.82	0-7	0	0-0	0	0-0	0	0-0	0	0-0
MA0051	IRF2	TRP-CLUSTER	0	0	0	0	0	0	0	0	0	0	0	0	0	0	0	0
MA0052	MEF2A	MADS	0	0	0	0	0	0	0	0	0	0	0	0	0	0	0	0
MA0053	MNB1A	ZN-FINGER, DOF	2.31	0-8	2.70	0-9	2.68	0-9	2.56	0-8.06	0	0-0	0	0-0	0	0-0	2.51	0-9.01
MA0054	MYB.ph3	TRP-CLUSTER	0	0	0	0	0	0	0	0	0	0	0	0	0	0	0	0
MA0055	Myf	bHLH	0	0	0	0	0	0	0	0	0	0	0	0	0	0	0	0
MA0056	ZNF42_1-4	ZN-FINGER, C2H2	0	0-0	3.68	0-11	4.06	0-10	3.90	0-11.06	0	0-0	0	0-0	0	0-0	0	0-0
MA0057	ZNF42_5-13	ZN-FINGER, C2H2	2.54	0-8	2.55	0-8	0	0-0	0	0-0	0	0-0	0	0-0	0	0-0	0	0-0
MA0058	MAX	bHLH-ZIP	0	0	0	0	0	0	0	0	0	0	0	0	0	0	0	0
MA0059	MYC-MAX	bHLH-ZIP	0	0	0	0	0	0	0	0	0	0	0	0	0	0	0	0
MA0060	NF-Y	CAAT-BOX	0	0	0	0	0	0	0	0	0	0	0	0	0	0	0	0
MA0061	NF-kappaB	REL	0	0	0	0	0	0	0	0	0	0	0	0	0	0	0	0
MA0062	GABPA	ETS	0	0-0	1.55	0-5	1.67	0-5	0	0-0	0	0-0	0	0-0	0	0-0	0	0-0
MA0063	Nkx2-5	HOMEO	0	0-0	1.07	0-5	0	0-0	1.12	0-5.06	0	0-0	0	0-0	0	0-0	0	0-0
MA0064	PBF	ZN-FINGER, DOF	1.97	0-7	2.44	0-9	2.42	0-8.01	2.36	0-8	1.97	0-7	0	0-0	0	0-0	2.15	0-9.01
MA0065	PPARG-RXRA	NUCLEAR RECEPTOR	0	0	0	0	0	0	0	0	0	0	0	0	0	0	0	0
MA0066	PPARG	NUCLEAR RECEPTOR	0	0-0	0	0-0	0	0-0	0	0-0	0	0-0	0	0-0	0	0-0	0.44	0-3
MA0067	Pax2	PAIRED	0	0	0	0	0	0	0	0	0	0	0	0	0	0	0	0
MA0068	Pax4	PAIRED-HOMEO	0	0	0	0	0	0	0	0	0	0	0	0	0	0	0	0
MA0069	Pax6	PAIRED	0	0	0	0	0	0	0	0	0	0	0	0	0	0	0	0
MA0070	Pbx	HOMEO	0	0	0	0	0	0	0	0	0	0	0	0	0	0	0	0
MA0071	RORA	NUCLEAR RECEPTOR	0	0	0	0	0	0	0	0	0	0	0	0	0	0	0	0
MA0072	RORA1	NUCLEAR RECEPTOR	0	0	0	0	0	0	0	0	0	0	0	0	0	0	0	0
MA0073	RREB1	ZN-FINGER, C2H2	0	0-0	0	0-0	0	0-0	3.90	0-21.42	0	0-0	0	0-0	0	0-0	0	0-0
MA0074	RXR-VDR	NUCLEAR RECEPTOR	0	0	0	0	0	0	0	0	0	0	0	0	0	0	0	0
MA0075	Prrx2	HOMEO	0	0-0	2.67	0-12	0	0-0	3.16	0-12.12	0	0-0	0	0-0	0	0-0	0	0-0
MA0076	ELK4	ETS	0	0-0	0	0-0	0.565	0-3	0	0-0	0	0-0	0	0-0	0	0-0	0	0-0
MA0077	SOX9	HMG	0	0	0	0	0	0	0	0	0	0	0	0	0	0	0	0
MA0078	Sox17	HMG	0	0	0	0	0	0	0	0	0	0	0	0	0	0	0	0
MA0079	SP1	ZN-FINGER, C2H2	0	0-0	3.19	0-9.72	0	0-0	3.39	0-9	0	0-0	3.45	0-10	0	0-0	3.52	0-10
MA0080	SPI1	ETS	3.18	0-7	3.70	0-10	3.71	0-10	3.44	0-10	0	0-0	0	0-0	0	0-0	0	0-0
MA0081	SPIB	ETS	0	0-0	3.78	0-9	3.94	0-10	3.56	0-9	0	0-0	0	0-0	0	0-0	0	0-0
MA0082	SQUA	MADS	0	0-0	2.70	0-10	0	0-0	3.03	0-11.12	0	0-0	0	0-0	0	0-0	0	0-0
MA0083	SRF	MADS	0	0	0	0	0	0	0	0	0	0	0	0	0	0	0	0
MA0084	SRY	HMG	0	0-0	2.15	0-7.72	0	0-0	2.35	0-7.06	0	0-0	0	0-0	0	0-0	0	0-0
MA0085	SU_h	IPT/TIG domain	0	0-0	0	0-0	0	0-0	1.35	0-4.06	0	0-0	0	0-0	0	0-0	0	0-0
MA0086	Snail	ZN-FINGER, C2H2	0	0	0	0	0	0	0	0	0	0	0	0	0	0	0	0
MA0087	Sox5	HMG	0	0	0	0	0	0	0	0	0	0	0	0	0	0	0	0
MA0088	Staf	ZN-FINGER, C2H2	0	0-0	0	0-0	0	0-0	0	0-0	2.52	0-7	2.50	0-8	0	0-0	2.59	0-8
MA0089	TCF11-MafG	bZIP	1.66	0-6	0	0-0	0	0-0	0	0-0	0	0-0	0	0-0	0	0-0	0	0-0

MA0090	TEAD	TEA	1.69	0-5.56	0	0-0	0	0-0	0	0-0	0	0-0	0	0-0	0	0-0	0	0-0
MA0091	TAL1-TCF3	bHLH	0	0	0	0	0	0	0	0	0	0	0	0	0	0	0	0
MA0092	HAND1-TCF3	bHLH	0	0-0	0	0-0	0	0-0	0	0-0	0	0-0	0	0-0	0	0-0	2.57	0-7
MA0093	USF1	bHLH-ZIP	0	0	0	0	0	0	0	0	0	0	0	0	0	0	0	0
MA0094	Ubx	HOMEO	7.00	0-0	0.10	0-4	0	0-0	0.09	0-2.18	0	0-0	0	0-0	0	0-0	5.00	0-0
MA0095	YY1	ZN-FINGER, C2H2	0	0-0	0	0-0	0	0-0	0	0-0	2.15	0-6	0	0-0	0	0-0	0	0-0
MA0096	bZIP910	bZIP	0	0	0	0	0	0	0	0	0	0	0	0	0	0	0	0
MA0097	bZIP911	bZIP	0	0	0	0	0	0	0	0	0	0	0	0	0	0	0	0
MA0098	c-ETS	ETS	0	0-0	1.09	0-5.72	0.99	0-5	0	0-0	0	0-0	0	0-0	0	0-0	0	0-0
MA0099	Fos	bZIP	2.21	0-7	2.27	0-7	2.26	0-7	0	0-0	0	0-0	0	0-0	2.51	0-7	2.41	0-8
MA0100	Myb	TRP-CLUSTER	0	0	0	0	0	0	0	0	0	0	0	0	0	0	0	0
MA0101	REL	REL	0	0	0	0	0	0	0	0	0	0	0	0	0	0	0	0
MA0102	cEBP	bZIP	0	0-0	0	0-0	0	0-0	1.58	0-6	0	0-0	0	0-0	0	0-0	0	0-0
MA0103	deltaEF1	ZN-FINGER, C2H2	0	0	0	0	0	0	0	0	0	0	0	0	0	0	0	0
MA0104	Mycn	bHLH-ZIP	0	0	0	0	0	0	0	0	0	0	0	0	0	0	0	0
MA0105	NFKB1	REL	0	0	0	0	0	0	0	0	0	0	0	0	0	0	0	0
MA0106	TP53	P53	0	0	0	0	0	0	0	0	0	0	0	0	0	0	0	0
MA0107	RELA	REL	0	0	0	0	0	0	0	0	0	0	0	0	0	0	0	0
MA0108	TBP	TATA-box	0	0	0	0	0	0	0	0	0	0	0	0	0	0	0	0
MA0109	RUSH1-alfa	ZN-FINGER, GATA	0.52	0-3	0.63	0-3	0	0-0	0	0-0	0	0-0	0.57	0-3	0	0-0	0	0-0
MA0110	ATHB5	HOMEO-ZIP	0	0	0	0	0	0	0	0	0	0	0	0	0	0	0	0
MA0111	Spz1	bHLH-ZIP	0	0-0	0	0-0	0	0-0	0	0-0	0	0-0	1.99	0-6	2.10	0-5.01	2.06	0-5.01
MA0112	ESR1	NUCLEAR	0	0	0	0	0	0	0	0	0	0	0	0	0	0	0	0
MA0113	NR3C1	NUCLEAR	0	0	0	0	0	0	0	0	0	0	0	0	0	0	0	0
MA0114	HNF4	NUCLEAR	0	0	0	0	0	0	0	0	0	0	0	0	0	0	0	0
MA0115	NR1H2-RXR	NUCLEAR RECEPTOR	0	0-0	0	0-0	0	0-0	0.08	0-1	0	0-0	0	0-0	0	0-0	0	0-0
MA0116	Roaz	ZN-FINGER, C2H2	0	0	0	0	0	0	0	0	0	0	0	0	0	0	0	0
MA0117	MafB	bZIP, MAF	1.64	0-5	0	0-0	0	0-0	0	0-0	0	0-0	0	0-0	0	0-0	0	0-0
MA0118	Macho-1	ZN-FINGER, C2H2	0	0	0	0	0	0	0	0	0	0	0	0	0	0	0	0
MA0119	Hox11-CTF1	HOMEO/CAAT	0	0	0	0	0	0	0	0	0	0	0	0	0	0	0	0
MA0120	ID1	ZN-FINGER, C2H2	0	0-0	3.07	0-20.72	0	0-0	3.05	0-20.18	0	0-0	0	0-0	0	0-0	0	0-0
MA0121	ARR10	TRP-CLUSTER	0	0	0	0	0	0	0	0	0	0	0	0	0	0	0	0
MA0122	Bapx1	HOMEO	2.64	0-7	2.69	0-7	0	0-0	0	0-0	2.77	0-7	2.89	0-8	2.76	0-7	2.80	0-7
MA0123	ABI4	AP2	0	0-0	2.08	0-10	0	0-0	0	0-0	0	0-0	0	0-0	0	0-0	0	0-0

Distribution of JASPAR motifs (average number of motifs found enriched per sequence and 1st to 99th percentile range) in sequences flanking (+/- 1,000 bp) the integration sites of MLV, HIV and HIVmIN vectors in <u>Hela cells</u> (see Figure 1 for vector identification).

Matrix ID	TF name	TF class	MLV		HIV		HIVmIN	
			mean	1-99th percentile	mean	1-99th percentile	mean	1-99th percentile
MA0001	AGL3	MADS	0	0	0	0	0	0
MA0002	RUNX1	RUNT	0	0	0	0	0	0
MA0003	TFAP2A	AP2	3.22	0-11	0	0-0	2.89	0-9
MA0004	Arnt	bHLH	0	0	0	0	0	0
MA0005	Agamous	MADS	0	0	0	0	0	0
MA0006	Arnt-Ahr	bHLH	0	0	0	0	0	0
MA0007	Ar	NUCLEAR RECEPTOR	0	0	0	0	0	0

MA0008	Athb-1	HOMEO-ZIP	0	0	0	0	0	0
MA0009	T	T-BOX	0	0	0	0	0	0
MA0010	Broad-complex_1	ZN-FINGER, C2H2	0	0-0	0	0-0	4.76	0-29.76
MA0011	Broad-complex_2	ZN-FINGER, C2H2	0	0	0	0	0	0
MA0012	Broad-complex_3	ZN-FINGER, C2H2	1.93	0-13.37	0	0-0	2.05	0-13.52
MA0013	Broad-complex_4	ZN-FINGER, C2H2	2.73	0-25	0	0-0	2.97	0-23.52
MA0014	Pax5	PAIRED	0	0	0	0	0	0
MA0015	CF2-II	ZN-FINGER, C2H2	0	0	0	0	0	0
MA0016	CFI-USP	NUCLEAR RECEPTOR	0	0	0	0	0	0
MA0017	NR2F1	NUCLEAR RECEPTOR	0	0	0	0	0	0
MA0018	CREB1	bZIP	0	0	0	0	0	0
MA0019	Chop-cEBP	bZIP	0	0-0	3.09	0-10	0	0-0
MA0020	Dof2	ZN-FINGER, DOF	3.89	0-10	0	0-0	0	0-0
MA0021	Dof3	ZN-FINGER, DOF	3.39	0-9	0	0-0	3.44	0-9
MA0022	Dorsal_1	REL	0	0-0	0	0-0	1.88	0-6
MA0023	Dorsal_2	REL	0	0	0	0	0	0
MA0024	E2F1	Unknown	0	0	0	0	0	0
MA0025	E74A	ETS	0	0	0	0	0	0
MA0026	E74A	ETS	2.18	0-7	0	0-0	1.98	0-7
MA0027	En1	HOMEO	0	0	0	0	0	0
MA0028	ELK1	ETS	0	0	0	0	0	0
MA0029	Evi1	ZN-FINGER, C2H2	0	0	0	0	0	0
MA0030	FOXF2	FORKHEAD	0	0	0	0	0	0
MA0031	FOXD1	FORKHEAD	1.39	0-5	0	0-0	0	0-0
MA0032	FOXC1	FORKHEAD	0	0	0	0	0	0
MA0032	FOXC1	FORKHEAD	0	0	0	0	0	0
MA0033	FOXL1	FORKHEAD	0	0	0	0	0	0
MA0034	GAMYB	TRP-CLUSTER	0	0	0	0	0	0
MA0035	Gata1	ZN-FINGER, GATA	0	0	0	0	0	0
MA0036	GATA2	ZN-FINGER, GATA	0	0	0	0	0	0
MA0037	GATA3	ZN-FINGER, GATA	0	0	0	0	0	0
MA0038	Gfi	ZN-FINGER, C2H2	2.29	0-6	0	0-0	0	0-0
MA0040	Foxq1	FORKHEAD	1.71	0-5.37	0	0-0	0	0-0
MA0041	Foxd3	FORKHEAD	4.04	0-25.74	0	0-0	4.49	0-22
MA0042	FOXI1	FORKHEAD	2.52	0-9	0	0-0	2.48	0-11
MA0043	HLF	bZIP	0	0	0	0	0	0
MA0044	HMG-1	HMG	0	0	0	0	0	0
MA0045	HMG-IY	HMG	0	0-0	0	0-0	6.99	0-36.52
MA0046	TCF1	HOMEO	0	0	0	0	0	0
MA0047	Foxa2	FORKHEAD	2.31	0-8	0	0-0	2.22	0-7
MA0048	NHLH1	bHLH	0	0	0	0	0	0
MA0049	Hunchback	ZN-FINGER, C2H2	5.89	0-36	0	0-0	6.64	0-36.76
MA0050	IRF1	TRP-CLUSTER	1.58	0-6	0	0-0	0	0-0
MA0051	IRF2	TRP-CLUSTER	0.82	0-4	0	0-0	0	0-0
MA0052	MEF2A	MADS	0	0	0	0	0	0
MA0053	MNB1A	ZN-FINGER, DOF	2.76	0-9.37	0	0-0	0	0-0
MA0054	MYB.ph3	TRP-CLUSTER	0	0	0	0	0	0
MA0055	Myf	bHLH	0	0	0	0	0	0
MA0056	ZNF42_1-4	ZN-FINGER, C2H2	3.68	0-11	0	0-0	0	0-0
MA0057	ZNF42_5-13	ZN-FINGER, C2H2	2.61	0-8.37	0	0-0	0	0-0

MA0058	MAX	bHLH-ZIP	1.06	0-5	0	0-0	0	0-0
MA0059	MYC-MAX	bHLH-ZIP	0.99	0-5	0	0-0	0	0-0
MA0060	NF-Y	CAAT-BOX	0	0	0	0	0	0
MA0061	NF-kappaB	REL	0	0	0	0	0	0
MA0062	GABPA	ETS	1.48	0-5	0	0-0	0	0-0
MA0063	Nkx2-5	HOMEO	1.04	0-5	0	0-0	0.97	0-5
MA0064	PBF	ZN-FINGER, DOF	2.47	0-9.37	2.00	0-6.69	0	0-0
MA0065	PPARG-RXRA	NUCLEAR RECEPTOR	0	0	0	0	0	0
MA0066	PPARG	NUCLEAR RECEPTOR	0	0	0	0	0	0
MA0067	Pax2	PAIRED	0	0	0	0	0	0
MA0068	Pax4	PAIRED-HOMEO	0	0	0	0	0	0
MA0069	Pax6	PAIRED	0	0	0	0	0	0
MA0070	Pbx	HOMEO	0	0	0	0	0	0
MA0071	RORA	NUCLEAR RECEPTOR	0	0	0	0	0	0
MA0072	RORA1	NUCLEAR RECEPTOR	0	0	0	0	0	0
MA0073	RREB1	ZN-FINGER, C2H2	3.21	0-20	0	0-0	0	0-0
MA0074	RXR-VDR	NUCLEAR RECEPTOR	0	0	0	0	0	0
MA0075	Prrx2	HOMEO	0	0-0	0	0-0	2.81	0-11.76
MA0076	ELK4	ETS	0.56	0-3	0	0-0	0	0-0
MA0077	SOX9	HMG	0	0	0	0	0	0
MA0078	Sox17	HMG	0	0	0	0	0	0
MA0079	SP1	ZN-FINGER, C2H2	3.28	0-10	3.67	0-10.69	3.29	0-9
MA0080	SPI1	ETS	3.56	0-10	0	0-0	0	0-0
MA0081	SPIB	ETS	3.50	0-9	0	0-0	0	0-0
MA0082	SQUA	MADS	0	0-0	0	0-0	2.99	0-12.76
MA0083	SRF	MADS	0	0	0	0	0	0
MA0084	SRY	HMG	2.19	0-8	0	0-0	2.21	0-8
MA0085	SU_h	IPT/TIG domain	0	0	0	0	0	0
MA0086	Snail	ZN-FINGER, C2H2	0	0	0	0	0	0
MA0087	Sox5	HMG	1.63	0-6	0	0-0	0	0-0
MA0088	Staf	ZN-FINGER, C2H2	0	0-0	2.67	0-7	0	0-0
MA0089	TCF11-MafG	bZIP	0	0	0	0	0	0
MA0090	TEAD	TEA	1.61	0-5	0	0-0	0	0-0
MA0091	TAL1-TCF3	bHLH	0	0	0	0	0	0
MA0092	HAND1-TCF3	bHLH	0	0	0	0	0	0
MA0093	USF1	bHLH-ZIP	0	0	0	0	0	0
MA0094	Ubx	HOMEO	0.11	0-5	0.01	0-0	0.08	0-2.76
MA0095	YY1	ZN-FINGER, C2H2	0	0	0	0	0	0
MA0096	bZIP910	bZIP	0.22	0-2	0	0-0	0	0-0
MA0097	bZIP911	bZIP	0	0	0	0	0	0
MA0098	c-ETS	ETS	1.11	0-5.37	0	0-0	0	0-0
MA0099	Fos	bZIP	2.84	0-8	0	0-0	2.47	0-7.76
MA0100	Myb	TRP-CLUSTER	0	0	0	0	0	0
MA0101	REL	REL	0	0	0	0	0	0
MA0102	cEBP	bZIP	0	0	0	0	0	0
MA0103	deltaEF1	ZN-FINGER, C2H2	0	0-0	3.85	0-10	0	0-0
MA0104	Mycn	bHLH-ZIP	0	0	0	0	0	0
MA0105	NFKB1	REL	0	0	0	0	0	0
MA0106	TP53	P53	0	0	0	0	0	0
MA0107	RELA	REL	0	0	0	0	0	0

MA0108	TBP	TATA-box	0	0	0	0	0	0
MA0109	RUSH1-alfa	ZN-FINGER, GATA	0	0	0	0	0	0
MA0110	ATHB5	HOMEO-ZIP	0	0	0	0	0	0
MA0111	Spz1	bHLH-ZIP	0	0-0	2.01	0-5	0	0-0
MA0112	ESR1	NUCLEAR	0	0-0	2.29	0-8.38	0	0-0
MA0113	NR3C1	NUCLEAR	0	0	0	0	0	0
MA0114	HNF4	NUCLEAR	0	0	0	0	0	0
MA0115	NR1H2-RXR	NUCLEAR RECEPTOR	0	0	0	0	0	0
MA0116	Roaz	ZN-FINGER, C2H2	0	0	0	0	0	0
MA0117	MafB	bZIP, MAF	0	0-0	1.62	0-5	0	0-0
MA0118	Macho-1	ZN-FINGER, C2H2	0	0	0	0	0	0
MA0119	Hox11-CTF1	HOMEO/CAAT	0.91	0-4	0	0-0	0	0-0
MA0120	ID1	ZN-FINGER, C2H2	3.01	0-24.37	0	0-0	3.39	0-25.76
MA0121	ARR10	TRP-CLUSTER	0	0	0	0	0	0
MA0122	Bapx1	HOMEO	0	0-0	0	0-7	2.64	0-7
MA0123	ABI4	AP2	0	0	0	0	0	0
