# Supplementary material for: Transcription Factor Binding Sites Are Genetic Determinants of Retroviral Integration in the Human Genome
Source: PLoS One. 2009 Feb 24;4(2):e4571. doi: 10.1371/journal.pone.0004571 (PMC2642719; doi:10.1371/journal.pone.0004571)
Supplement: Table S4 — (0.05 MB PDF) [file pone.0004571.s007.pdf]

Supplementary Table 4

Statistical significance of evolutionarily conserved TFBS analysis. A Fisher's exact test was applied to compare the percentage of sequences containing at least one conserved TFBS between experimental datasets and corresponding fitted and random backgrounds.

CD34$^+$ HSC cells

	fitted background	random
**Controls**	5.8e-05	9.7e-06
**MLV**	6.4e-20	1.5e-33
**ΔU3-MLV**	0.178	0.026
**SFFV-MLV**	2.1e-04	2.1e-04
**HIV**	0.511	0.023
**ΔU3-HIV[CMV]**	0.097	2.3e-04
**ΔU3-HIV[MLV]**	0.475	0.068
**MLV-HIV**	1.4e-03	9.3e-08

Hela cells

	fitted background	random
**MLV**	4.1e-18	3.3e-30
**HIV**	0.053	3.1e-06
**HIVmIN**	1.4e-06	6.8e-10
