# Supplementary material for: Transcription Factor Binding Sites Are Genetic Determinants of Retroviral Integration in the Human Genome
Source: PLoS One. 2009 Feb 24;4(2):e4571. doi: 10.1371/journal.pone.0004571 (PMC2642719; doi:10.1371/journal.pone.0004571)
Supplement: Table S5 — (0.04 MB PDF) [file pone.0004571.s008.pdf]

Supplementary Table 5

Distribution of TRANSFAC conserved matrices (total counts) in sequences flanking (+/- 1,000 bp) the integration sites of different MLV and HIV vectors in human CD34$^+$ HSCs (see Figure 1 for vector identification).
*** p<0.05, Fisher test over matched background.**

Matrix	AccNumb	Binding Factors	Controls	MLV	ΔU3-MLV	SFFV-MLV	HIV	ΔU3-HIV[CMV]	ΔU3-HIV[MLV]	MLV-HIV
M00002	V$E47_01	E47	0	0	0	0	0	0	0	0
M00005	V$AP4_01	AP-4	0	0	0	0	0	0	0	0
M00006	V$MEF2_01	MEF-2A	*11	22	0	4	6	*12	*6	2
M00007	V$ELK1_01	Elk-1	0	0	0	*2	0	0	0	0
M00017	V$ATF_01	ATF	0	2	0	2	0	0	0	0
M00024	V$E2F_01	E2F	0	*8	0	0	0	0	0	0
M00025	V$ELK1_02	Elk-1	0	4	0	0	0	0	0	0
M00026	V$RSRFC4_01	RSRFC4	7	6	0	*6	0	6	0	2
M00033	V$P300_01	p300	0	0	0	0	0	0	0	0
M00034	V$P53_01	p53	0	2	*4	0	0	0	0	0
M00037	V$NFE2_01	NF-E2	2	12	0	2	0	0	0	2
M00039	V$CREB_01	CREB, deltaCREB	0	2	0	2	0	0	0	0
M00040	V$CREBP1_01	ATF-2	1	6	2	*6	0	0	0	0
M00041	V$CREBP1CJUN_01	ATF-2, c-Jun	0	4	2	0	0	0	2	0
M00045	V$E4BP4_01	E4BP4	6	12	2	*10	*10	*14	2	4
M00050	V$E2F_02	E2F, E2F-1, E2F-2, E2F-3a, E2F-4, E2F-5	0	4	2	0	0	0	0	0
M00051	V$NFKAPPAB50_01	NF-kappaB1	0	0	0	0	2	0	0	0
M00052	V$NFKAPPAB65_01	RelA	2	4	0	0	0	*6	0	2
M00053	V$CREL_01	c-Rel	0	6	2	0	0	*8	0	0
M00054	V$NFKAPPAB_01	NF-kappaB, NF-kappaB1, RelA	0	0	0	0	0	4	0	0
M00056	V$MYOGNF1_01	NF-1	0	4	2	0	0	0	0	0
M00059	V$YY1_01	YY1	3	2	0	0	0	2	0	0
M00062	V$IRF1_01	IRF-1	4	*20	0	4	4	2	2	6
M00065	V$TAL1BETAE47_01	E47, Tal-1beta	2	*8	0	0	0	0	0	0
M00066	V$TAL1ALPHAE47_01	E47, Tal-1	2	8	0	0	0	2	0	4
M00069	V$YY1_02	YY1	2	*12	0	0	0	0	0	2
M00070	V$TAL1BETAITF2_01	ITF-2, Tal-1beta	1	*12	2	0	0	2	0	4
M00071	V$E47_02	E47	0	0	0	0	0	0	0	0
M00076	V$GATA2_01	GATA-2	0	0	0	0	0	0	0	0
M00077	V$GATA3_01	GATA-3	2	0	0	0	0	0	0	0
M00084	V$MZF1_02	MZF-1	0	2	0	0	0	0	0	0
M00085	V$ZID_01	ZID	3	4	0	2	0	0	0	0
M00095	V$CDP_01	CUTL1	4	12	2	2	4	4	2	6

Matrix	AccNumb	Binding Factors	Controls	MLV	ΔU3-MLV	SFFV-MLV	HIV	ΔU3-HIV[CMV]	ΔU3-HIV[MLV]	MLV-HIV
M00096	V$PBX1_01	Pbx1a	2	10	0	*4	0	4	0	*6
M00097	V$PAX6_01	Pax-6	5	8	0	*6	2	2	0	0
M00098	V$PAX2_01	Pax-2	0	4	0	0	0	0	0	0
M00102	V$CDP_02	CUTL1	*23	16	4	6	12	8	6	14
M00104	V$CDPCR1_01	CUTL1	1	2	0	0	0	0	0	0
M00105	V$CDPCR3_01	CUTL1	7	10	0	2	2	4	0	0
M00106	V$CDPCR3HD_01	CUTL1	4	0	0	0	0	2	0	2
M00109	V$CEBPB_01	C/EBPbeta	2	4	0	0	2	4	2	0
M00113	V$CREB_02	CREB, deltaCREB	0	2	0	0	0	0	0	0
M00114	V$TAXCREB_01	CREB, deltaCREB	0	2	0	0	0	0	0	0
M00115	V$TAXCREB_02	CREB, deltaCREB	2	4	0	0	0	0	0	0
M00116	V$CEBPA_01	C/EBPalpha	3	4	2	0	2	2	2	2
M00117	V$CEBPB_02	C/EBPbeta	1	10	0	0	0	0	0	0
M00118	V$MYCMAX_01	c-Myc, Max1	0	2	2	0	0	0	2	0
M00119	V$MAX_01	Max1	0	0	*2	0	0	0	0	0
M00121	V$USF_01	USF1	0	2	*4	2	0	0	0	0
M00122	V$USF_02	USF1	0	0	0	0	0	0	0	0
M00123	V$MYCMAX_02	c-Myc, Max1	0	0	0	0	0	0	0	0
M00124	V$PBX1_02	Pbx1a	3	10	0	4	6	4	2	8
M00126	V$GATA1_02	GATA-1	*7	0	2	0	0	2	0	2
M00127	V$GATA1_03	GATA-1	0	6	2	0	0	0	0	2
M00128	V$GATA1_04	GATA-1	1	4	0	2	2	0	0	0
M00130	V$FOXD3_01	FOXD3	*6	6	0	2	0	0	0	4
M00132	V$HNF1_01	HNF-1A	*9	12	2	2	2	2	4	0
M00133	V$TST1_01	POU3F1	3	*12	0	0	2	4	0	0
M00134	V$HNF4_01	HNF-4alpha2	0	2	0	0	0	0	0	0
M00135	V$OCT1_01	POU2F1	6	*20	*6	2	2	4	2	6
M00136	V$OCT1_02	POU2F1	9	12	0	*8	*12	10	4	8
M00137	V$OCT1_03	POU2F1	5	2	0	2	2	4	0	0
M00138	V$OCT1_04	POU2F1	*5	8	*8	0	0	2	0	*8
M00143	V$PAX5_01	Pax-5	0	0	0	0	0	0	0	0
M00144	V$PAX5_02	Pax-5	0	2	0	0	0	2	0	2
M00145	V$BRN2_01	POU3F2	*12	16	0	0	6	8	0	8
M00146	V$HSF1_01	HSF1 (long)	4	4	0	2	2	2	2	2
M00147	V$HSF2_01	HSF2	2	8	0	2	0	0	2	0
M00152	V$SRF_01	SRF	1	2	0	0	2	4	0	4
M00155	V$ARP1_01	ARP-1	0	8	2	2	2	0	0	0
M00156	V$RORA1_01	RORalpha1	*4	0	0	0	2	2	0	0
M00157	V$RORA2_01	RORalpha2	*11	12	2	0	*12	*12	0	2
M00158	V$COUP_01	COUP-TF1, HNF-4alpha2	0	0	0	2	0	0	0	0

Matrix	AccNumb	Binding Factors	Controls	MLV	ΔU3-MLV	SFFV-MLV	HIV	ΔU3-HIV[CMV]	ΔU3-HIV[MLV]	MLV-HIV
M00159	V$CEBP_01	C/EBPalpha	0	0	0	0	0	0	0	0
M00160	V$SRY_02	SRY	2	4	0	2	0	0	2	2
M00161	V$OCT1_05	POU2F1	7	16	6	0	4	8	0	*14
M00162	V$OCT1_06	POU2F1	*4	8	0	0	2	0	0	4
M00172	V$AP1FJ_Q2	AP-1, c-Fos, c-Jun	0	0	0	0	0	0	0	0
M00173	V$AP1_Q2	AP-1	0	2	2	0	0	0	0	0
M00174	V$AP1_Q6	AP-1	0	0	0	0	0	0	0	0
M00177	V$CREB_Q2	CREB	0	8	0	2	0	0	0	0
M00178	V$CREB_Q4	CREB	0	*8	2	0	0	0	0	0
M00179	V$CREBP1_Q2	ATF-2	0	8	2	2	0	0	0	0
M00183	V$MYB_Q6	c-Myb	1	8	0	0	*4	0	0	0
M00185	V$NFY_Q6	CP1A, CP1C, NF-Y, NF-YA	0	0	0	0	0	0	0	0
M00186	V$SRF_Q6	SRF	1	2	0	0	*6	0	0	0
M00187	V$USF_Q6	USF1	0	0	0	2	0	0	0	0
M00188	V$AP1_Q4	AP-1	2	8	0	0	0	0	0	0
M00189	V$AP2_Q6	AP-2alphaA, AP-2gamma	0	0	0	0	0	0	0	0
M00190	V$CEBP_Q2	C/EBPalpha	0	6	0	0	0	0	0	2
M00191	V$ER_Q6	ER-alpha	0	4	0	0	0	0	0	0
M00192	V$GR_Q6	GR-alpha, GR-beta	0	4	0	0	2	0	0	0
M00193	V$NF1_Q6	NF-1	0	0	0	0	0	0	0	0
M00194	V$NFKB_Q6	NF-kappaB, NF-kappaB1	0	2	0	0	0	0	0	0
M00195	V$OCT1_Q6	POU2F1	6	4	2	2	4	2	2	0
M00201	V$CEBP_C	C/EBPalpha	2	4	0	0	2	2	0	4
M00203	V$GATA_C	GATA-1, GATA-2, GATA-3	*3	4	2	0	0	0	2	2
M00205	V$GRE_C	GR-alpha	2	10	0	2	2	0	0	0
M00206	V$HNF1_C	HNF-1A	6	12	0	0	2	2	0	6
M00208	V$NFKB_C	NF-kappaB, NF-kappaB1, NF-kappaB2	0	2	0	0	0	0	2	0
M00209	V$NFY_C	CP1A, NF-Y, NF-YA	0	*12	0	2	0	0	0	2
M00210	V$OCT_C	Oct-B1, oct-B2, oct-B3, POU2F1, POU2F2, POU2F2(Oct-2.1), POU2F2B, POU2F2C	10	8	6	2	8	8	2	8
M00215	V$SRF_C	SRF	0	0	2	0	0	2	0	2
M00216	V$TATA_C	TBP, TFIID	3	10	4	0	4	0	0	2
M00220	V$SREBP1_01	SREBP-1a, SREBP-1b, SREBP-1c	0	*6	0	*4	0	2	0	0
M00221	V$SREBP1_02	SREBP-1a, SREBP-1b, SREBP-1c	2	0	0	2	0	0	0	0
M00222	V$HAND1E47_01	E47	0	2	2	0	0	0	0	4
M00223	V$STAT_01	STAT1alpha, STAT1beta, STAT2, STAT3, STAT4, STAT6	1	2	2	2	2	2	0	0
M00224	V$STAT1_01	STAT1alpha, STAT1beta	0	2	2	2	0	2	0	0
M00225	V$STAT3_01	STAT3	0	*8	*4	0	0	2	0	2
M00231	V$MEF2_02	MEF-2A	3	*8	0	*4	0	4	0	0
M00232	V$MEF2_03	MEF-2A	6	4	0	0	0	4	0	0

Matrix	AccNumb	Binding Factors	Controls	MLV	ΔU3-MLV	SFFV-MLV	HIV	ΔU3-HIV[CMV]	ΔU3-HIV[MLV]	MLV-HIV
M00233	V$MEF2_04	MEF-2A	*7	12	4	0	2	2	4	4
M00235	V$AHRARNT_01	AhR, Arnt	0	*8	2	0	0	0	0	0
M00236	V$ARNT_01	Arnt	0	2	*2	2	0	0	0	0
M00237	V$AHRARNT_02	AhR, Arnt	0	0	0	0	0	0	0	0
M00243	V$EGR1_01	Egr-1	0	2	0	0	0	2	0	0
M00245	V$EGR3_01	Egr-3	0	4	0	0	0	2	0	0
M00246	V$EGR2_01	Egr-2	0	4	0	2	0	2	0	0
M00248	V$OCT1_07	POU2F1	12	18	4	6	6	*16	4	*14
M00249	V$CHOP_01	C/EBPalpha, CHOP-10	0	4	0	0	2	2	0	2
M00251	V$XBP1_01	XBP-1	0	4	2	0	0	0	0	0
M00252	V$TATA_01	TBP	1	0	0	0	0	0	0	0
M00256	V$NRSF_01	NRSF form 1, NRSF form 2	1	0	2	0	0	2	0	0
M00257	V$RREB1_01	RREB-1	2	6	0	2	0	0	0	2
M00258	V$ISRE_01	ISGF-3	1	20	4	2	2	0	2	2
M00260	V$HLF_01	Hlf	3	10	0	0	2	8	0	2
M00272	V$P53_02	p53	0	0	0	0	0	0	0	0
M00277	V$LMO2COM_01	Lmo2	0	0	0	0	0	0	0	0
M00278	V$LMO2COM_02	Lmo2	5	*18	0	4	2	0	0	0
M00279	V$MIF1_01	MIF-1	0	*8	0	2	2	0	0	0
M00280	V$RFX1_01	RFX1	*4	*8	0	2	2	2	2	0
M00281	V$RFX1_02	RFX1	0	*14	2	0	2	6	0	4
M00284	V$TCF11MAFG_01	LCR-F1	3	*12	*6	2	0	2	2	4
M00285	V$TCF11_01	LCR-F1	1	0	0	0	0	0	0	0
M00287	V$NFY_01	NF-Y	0	0	0	0	0	0	0	0
M00289	V$HFH3_01	FOXI1	2	*8	0	2	2	4	0	0
M00290	V$FREAC2_01	FOXF2	2	*20	0	2	2	4	2	6
M00291	V$FREAC3_01	FOXC1	*7	*20	0	0	4	4	2	4
M00292	V$FREAC4_01	FOXD1	3	*20	0	0	4	0	0	4
M00293	V$FREAC7_01	FOXL1	6	*14	0	4	6	6	2	6
M00302	V$NFAT_Q6	NF-AT1, NF-AT2, NF-AT3, NF-AT4	0	0	0	0	0	0	0	0
M00346	V$GATA1_05	GATA-1	4	18	2	0	4	4	0	6
M00410	V$SOX9_B1	Sox9	0	*12	0	2	0	0	0	4
M00412	V$AREB6_01	AREB6	0	2	0	0	2	0	0	0
M00413	V$AREB6_02	AREB6	0	0	0	0	0	0	0	0
M00414	V$AREB6_03	AREB6	0	4	*4	0	0	0	2	2
M00416	V$CART1_01	Cart-1	*14	10	6	2	10	14	2	10
M00418	V$TGIF_01	TGIF	1	4	0	0	0	0	0	2
M00419	V$MEIS1_01	Meis-1	0	2	0	0	0	0	0	0
M00420	V$MEIS1AHOXA9_01	HOXA9B, Meis-1	9	12	2	0	0	2	4	10
M00421	V$MEIS1BHOXA9_02	HOXA9B, Meis-1	7	4	2	0	0	4	2	8

Matrix	AccNumb	Binding Factors	Controls	MLV	ΔU3-MLV	SFFV-MLV	HIV	ΔU3-HIV[CMV]	ΔU3-HIV[MLV]	MLV-HIV
M00422	V$FOXJ2_01	FOXJ2 (long isoform)	2	2	0	4	6	0	0	6
M00423	V$FOXJ2_02	FOXJ2 (long isoform)	10	14	6	0	14	10	4	16
M00424	V$NKX61_01	Nkx6-1	*11	14	0	0	2	4	0	*14
M00437	V$CHX10_01	Chx10	*10	10	4	4	2	10	0	*10
M00449	V$ZIC2_01	ZIC2	2	*8	0	0	0	0	0	0
M00451	V$NKX3A_01	Nkx3-1	3	4	2	2	2	2	2	6
M00453	V$IRF7_01	IRF-7A	3	*10	0	2	2	6	0	4
M00454	V$MRF2_01	MRF-2	4	*16	0	2	8	8	4	2
M00456	V$FAC1_01	FAC1	*6	*12	0	*6	0	4	0	4
M00457	V$STAT5A_01	STAT5A	2	*14	0	0	*6	2	0	0
M00459	V$STAT5B_01	STAT5B	2	*14	0	2	6	4	0	0
M00460	V$STAT5A_02	STAT5A	0	*10	2	0	2	*6	0	0
M00463	V$POU3F2_01	POU3F2 (N-Oct-5a), POU3F2 (N-Oct-5b)	7	16	2	2	0	8	2	8
M00464	V$POU3F2_02	POU3F2 (N-Oct-5a), POU3F2 (N-Oct-5b)	*25	22	4	4	10	16	6	12
M00472	V$FOXO4_01	FOXO4	2	2	2	0	2	2	0	2
M00474	V$FOXO1_02	FOXO1a	0	30	2	2	6	4	4	6
M00476	V$FOXO4_02	FOXO4	7	*20	4	2	4	4	2	4
M00477	V$FOXO3_01	FOXO3a, FOXO3b	9	*30	2	2	6	10	0	8
M00478	V$CDC5_01	Cdc5	9	16	2	2	4	8	4	6
M00480	V$LUN1_01	LUN-1	0	6	*4	0	0	0	0	0
M00483	V$ATF6_01	ATF6	0	0	0	0	0	2	0	0
M00484	V$NCX_01	NCX	3	*14	0	2	2	4	0	4
M00485	V$NKX22_01	Nkx2-2	5	12	2	2	0	4	0	8
M00490	V$BACH2_01	Bach2	3	4	2	2	0	0	0	4
M00491	V$MAZR_01	MAZR	0	0	0	0	0	0	0	0
M00495	V$BACH1_01	Bach1	2	*14	0	2	2	0	0	6
M00510	V$LHX3_01	LHX3a, LHX3b	5	2	2	0	4	2	2	8
M00512	V$PPARG_01	PPAR-gamma1, PPAR-gamma2	2	4	0	2	2	0	0	0
M00515	V$PPARG_02	PPAR-gamma1, PPAR-gamma2	0	*6	0	0	0	0	0	0
M00516	V$E2F_03	E2F	0	2	0	0	0	0	0	0
M00517	V$AP1_01	AP-1, c-Fos, Fra-1, JunB, JunD	0	4	0	2	0	0	0	2
M00526	V$GCNF_01	GCNF-1, GCNF-2	4	4	0	4	0	0	2	0
M00528	V$PPARG_03	PPAR-gamma1, PPAR-gamma2	0	4	0	0	0	0	0	0
M00532	V$RP58_01	RP58	2	6	0	0	0	2	0	2
M00539	V$ARNT_02	Arnt	0	4	2	0	0	0	0	0
M00615	V$MYCMAX_03	c-Myc, Max	0	*4	0	0	0	0	0	0

Distribution of TRANFAC conserved matrices (total counts) in sequences flanking (+/- 1,000 bp) the integration sites of MLV, HIV and HIVmIN vectors in Hela cells (see Figure 1 for vector identification).
*** p<0.05, Fisher test over matched background.**

Matrix	AccNumb	Binding Factors	MLV	HIV	HIVmIN
M00002	V$E47_01	E47	0	0	0
M00005	V$AP4_01	AP-4	0	0	0
M00006	V$MEF2_01	MEF-2A	12	4	2
M00007	V$ELK1_01	Elk-1	0	0	0
M00017	V$ATF_01	ATF	2	2	0
M00024	V$E2F_01	E2F	12	0	2
M00025	V$ELK1_02	Elk-1	0	0	0
M00026	V$RSRFC4_01	RSRFC4	2	4	4
M00033	V$P300_01	p300	0	0	0
M00034	V$P53_01	p53	4	2	*4
M00037	V$NFE2_01	NF-E2	24	2	0
M00039	V$CREB_01	CREB, deltaCREB	8	0	2
M00040	V$CREBP1_01	ATF-2	*26	0	2
M00041	V$CREBP1CJUN_01	ATF-2, c-Jun	24	0	4
M00045	V$E4BP4_01	E4BP4	*16	4	0
M00050	V$E2F_02	E2F, E2F-1, E2F-2, E2F-3a, E2F-4, E2F-5	4	0	2
M00051	V$NFKAPPAB50_01	NF-kappaB1	0	0	2
M00052	V$NFKAPPAB65_01	RelA	6	2	*4
M00053	V$CREL_01	c-Rel	*6	0	4
M00054	V$NFKAPPAB_01	NF-kappaB, NF-kappaB1, RelA	4	2	0
M00056	V$MYOGNF1_01	NF-1	0	0	0
M00059	V$YY1_01	YY1	14	2	2
M00062	V$IRF1_01	IRF-1	4	6	4
M00065	V$TAL1BETAE47_01	E47, Tal-1beta	4	0	0
M00066	V$TAL1ALPHAE47_01	E47, Tal-1	4	0	0
M00069	V$YY1_02	YY1	*20	0	8
M00070	V$TAL1BETAITF2_01	ITF-2, Tal-1beta	12	0	2
M00071	V$E47_02	E47	0	0	0
M00076	V$GATA2_01	GATA-2	0	0	0
M00077	V$GATA3_01	GATA-3	0	0	0
M00084	V$MZF1_02	MZF-1	0	2	0
M00085	V$ZID_01	ZID	4	0	0
M00095	V$CDP_01	CUTL1	12	0	4
M00096	V$PBX1_01	Pbx1a	0	10	2
M00097	V$PAX6_01	Pax-6	*16	6	2
M00098	V$PAX2_01	Pax-2	0	0	2
M00102	V$CDP_02	CUTL1	14	10	10
M00104	V$CDPCR1_01	CUTL1	2	0	0
M00105	V$CDPCR3_01	CUTL1	4	4	6
M00106	V$CDPCR3HD_01	CUTL1	2	0	2
M00109	V$CEBPB_01	C/EBPbeta	2	2	0
M00113	V$CREB_02	CREB, deltaCREB	0	0	0

Matrix	AccNumb	Binding Factors	MLV	HIV	HIVmIN
M00114	V$TAXCREB_01	CREB, deltaCREB	6	0	0
M00115	V$TAXCREB_02	CREB, deltaCREB	10	0	0
M00116	V$CEBPA_01	C/EBPalpha	2	0	2
M00117	V$CEBPB_02	C/EBPbeta	8	4	2
M00118	V$MYCMAX_01	c-Myc, Max1	0	0	0
M00119	V$MAX_01	Max1	0	0	*2
M00121	V$USF_01	USF1	0	0	2
M00122	V$USF_02	USF1	0	0	0
M00123	V$MYCMAX_02	c-Myc, Max1	0	0	0
M00124	V$PBX1_02	Pbx1a	10	0	4
M00126	V$GATA1_02	GATA-1	14	2	0
M00127	V$GATA1_03	GATA-1	4	2	4
M00128	V$GATA1_04	GATA-1	6	4	0
M00130	V$FOXD3_01	FOXD3	6	0	2
M00132	V$HNF1_01	HNF-1A	8	6	6
M00133	V$TST1_01	POU3F1	2	8	2
M00134	V$HNF4_01	HNF-4alpha2	4	0	2
M00135	V$OCT1_01	POU2F1	14	2	2
M00136	V$OCT1_02	POU2F1	6	2	8
M00137	V$OCT1_03	POU2F1	2	0	0
M00138	V$OCT1_04	POU2F1	8	0	0
M00143	V$PAX5_01	Pax-5	0	0	0
M00144	V$PAX5_02	Pax-5	0	0	0
M00145	V$BRN2_01	POU3F2	10	6	4
M00146	V$HSF1_01	HSF1 (long)	6	0	0
M00147	V$HSF2_01	HSF2	0	2	0
M00152	V$SRF_01	SRF	8	2	0
M00155	V$ARP1_01	ARP-1	6	0	0
M00156	V$RORA1_01	RORalpha1	2	4	2
M00157	V$RORA2_01	RORalpha2	10	8	2
M00158	V$COUP_01	COUP-TF1, HNF-4alpha2	6	2	2
M00159	V$CEBP_01	C/EBPalpha	0	0	0
M00160	V$SRY_02	SRY	8	8	0
M00161	V$OCT1_05	POU2F1	14	4	4
M00162	V$OCT1_06	POU2F1	8	8	0
M00172	V$AP1FJ_Q2	AP-1, c-Fos, c-Jun	0	2	0
M00173	V$AP1_Q2	AP-1	4	0	2
M00174	V$AP1_Q6	AP-1	0	2	0
M00177	V$CREB_Q2	CREB	6	0	2
M00178	V$CREB_Q4	CREB	8	0	0
M00179	V$CREBP1_Q2	ATF-2	14	0	0
M00183	V$MYB_Q6	c-Myb	0	2	0
M00185	V$NFY_Q6	CP1A, CP1C, NF-Y, NF-YA	0	0	0
M00186	V$SRF_Q6	SRF	4	2	0
M00187	V$USF_Q6	USF1	0	0	0
M00188	V$AP1_Q4	AP-1	4	2	0

Matrix	AccNumb	Binding Factors	MLV	HIV	HIVmIN
M00189	V$AP2_Q6	AP-2alphaA, AP-2gamma	0	0	0
M00190	V$CEBP_Q2	C/EBPalpha	4	2	2
M00191	V$ER_Q6	ER-alpha	0	2	0
M00192	V$GR_Q6	GR-alpha, GR-beta	4	0	0
M00193	V$NF1_Q6	NF-1	2	0	0
M00194	V$NFKB_Q6	NF-kappaB, NF-kappaB1	2	0	0
M00195	V$OCT1_Q6	POU2F1	8	2	2
M00201	V$CEBP_C	C/EBPalpha	8	2	0
M00203	V$GATA_C	GATA-1, GATA-2, GATA-3	6	0	0
M00205	V$GRE_C	GR-alpha	8	2	6
M00206	V$HNF1_C	HNF-1A	6	6	*8
M00208	V$NFKB_C	NF-kappaB, NF-kappaB1, NF-kappaB2	4	4	2
M00209	V$NFY_C	CP1A, NF-Y, NF-YA	2	2	0
M00210	V$OCT_C	Oct-B1, oct-B2, oct-B3, POU2F1, POU2F2, POU2F2(Oct-2.1), POU2F2B, POU2F2C	14	4	10
M00215	V$SRF_C	SRF	*10	0	2
M00216	V$TATA_C	TBP, TFIID	10	6	0
M00220	V$SREBP1_01	SREBP-1a, SREBP-1b, SREBP-1c	4	0	2
M00221	V$SREBP1_02	SREBP-1a, SREBP-1b, SREBP-1c	4	0	0
M00222	V$HAND1E47_01	E47	4	0	4
M00223	V$STAT_01	STAT1alpha, STAT1beta, STAT2, STAT3, STAT4, STAT6	*8	0	2
M00224	V$STAT1_01	STAT1alpha, STAT1beta	4	0	2
M00225	V$STAT3_01	STAT3	*8	0	0
M00231	V$MEF2_02	MEF-2A	*8	*6	2
M00232	V$MEF2_03	MEF-2A	4	2	2
M00233	V$MEF2_04	MEF-2A	12	8	2
M00235	V$AHRARNT_01	AhR, Arnt	4	2	0
M00236	V$ARNT_01	Arnt	0	0	0
M00237	V$AHRARNT_02	AhR, Arnt	2	2	0
M00243	V$EGR1_01	Egr-1	2	0	2
M00245	V$EGR3_01	Egr-3	2	0	0
M00246	V$EGR2_01	Egr-2	2	0	0
M00248	V$OCT1_07	POU2F1	*22	4	16
M00249	V$CHOP_01	C/EBPalpha, CHOP-10	6	2	0
M00251	V$XBP1_01	XBP-1	2	0	2
M00252	V$TATA_01	TBP	*10	0	0
M00256	V$NRSF_01	NRSF form 1, NRSF form 2	6	0	2
M00257	V$RREB1_01	RREB-1	*12	0	2
M00258	V$ISRE_01	ISGF-3	6	4	2
M00260	V$HLF_01	Hlf	4	4	0
M00272	V$P53_02	p53	0	0	0
M00277	V$LMO2COM_01	Lmo2	0	0	0
M00278	V$LMO2COM_02	Lmo2	2	4	0
M00279	V$MIF1_01	MIF-1	*8	2	*4
M00280	V$RFX1_01	RFX1	*10	2	2
M00281	V$RFX1_02	RFX1	2	0	6

Matrix	AccNumb	Binding Factors	MLV	HIV	HIVmIN
M00284	V$TCF11MAFG_01	LCR-F1	*20	6	2
M00285	V$TCF11_01	LCR-F1	0	0	0
M00287	V$NFY_01	NF-Y	0	0	0
M00289	V$HFH3_01	FOXI1	8	6	0
M00290	V$FREAC2_01	FOXF2	*32	*14	10
M00291	V$FREAC3_01	FOXC1	*16	6	8
M00292	V$FREAC4_01	FOXD1	*14	6	6
M00293	V$FREAC7_01	FOXL1	12	8	4
M00302	V$NFAT_Q6	NF-AT1, NF-AT2, NF-AT3, NF-AT4	2	0	0
M00346	V$GATA1_05	GATA-1	16	8	4
M00410	V$SOX9_B1	Sox9	6	0	2
M00412	V$AREB6_01	AREB6	2	0	0
M00413	V$AREB6_02	AREB6	0	0	0
M00414	V$AREB6_03	AREB6	4	2	0
M00416	V$CART1_01	Cart-1	16	2	8
M00418	V$TGIF_01	TGIF	0	0	2
M00419	V$MEIS1_01	Meis-1	0	0	0
M00420	V$MEIS1AHOXA9_01	HOXA9B, Meis-1	10	14	4
M00421	V$MEIS1BHOXA9_02	HOXA9B, Meis-1	6	10	4
M00422	V$FOXJ2_01	FOXJ2 (long isoform)	*16	2	2
M00423	V$FOXJ2_02	FOXJ2 (long isoform)	14	20	12
M00424	V$NKX61_01	Nkx6-1	10	10	0
M00437	V$CHX10_01	Chx10	14	6	2
M00449	V$ZIC2_01	ZIC2	2	4	0
M00451	V$NKX3A_01	Nkx3-1	6	4	2
M00453	V$IRF7_01	IRF-7A	6	6	0
M00454	V$MRF2_01	MRF-2	12	10	2
M00456	V$FAC1_01	FAC1	10	6	2
M00457	V$STAT5A_01	STAT5A	6	2	8
M00459	V$STAT5B_01	STAT5B	*14	4	10
M00460	V$STAT5A_02	STAT5A	*14	0	4
M00463	V$POU3F2_01	POU3F2 (N-Oct-5a), POU3F2 (N-Oct-5b)	8	8	4
M00464	V$POU3F2_02	POU3F2 (N-Oct-5a), POU3F2 (N-Oct-5b)	*38	8	8
M00472	V$FOXO4_01	FOXO4	0	0	2
M00474	V$FOXO1_02	FOXO1a	20	12	2
M00476	V$FOXO4_02	FOXO4	*24	6	2
M00477	V$FOXO3_01	FOXO3a, FOXO3b	28	14	4
M00478	V$CDC5_01	Cdc5	10	10	8
M00480	V$LUN1_01	LUN-1	4	0	2
M00483	V$ATF6_01	ATF6	*8	0	0
M00484	V$NCX_01	NCX	8	2	6
M00485	V$NKX22_01	Nkx2-2	4	4	6
M00490	V$BACH2_01	Bach2	*12	2	4
M00491	V$MAZR_01	MAZR	0	0	0
M00495	V$BACH1_01	Bach1	*34	6	4
M00510	V$LHX3_01	LHX3a, LHX3b	8	0	14

Matrix	AccNumb	Binding Factors	MLV	HIV	HIVmIN
M00512	V$PPARG_01	PPAR-gamma1, PPAR-gamma2	2	0	2
M00515	V$PPARG_02	PPAR-gamma1, PPAR-gamma2	0	0	0
M00516	V$E2F_03	E2F	0	0	0
M00517	V$AP1_01	AP-1, c-Fos, Fra-1, JunB, JunD	*10	0	0
M00526	V$GCNF_01	GCNF-1, GCNF-2	4	8	2
M00528	V$PPARG_03	PPAR-gamma1, PPAR-gamma2	4	0	2
M00532	V$RP58_01	RP58	4	2	2
M00539	V$ARNT_02	Arnt	6	0	2
M00615	V$MYCMAX_03	c-Myc, Max	2	0	0
